# Photobiomodulation suppresses JNK3 by activation of ERK/MKP7 to attenuate AMPA receptor endocytosis in Alzheimer's disease

**DOI:** 10.1111/acel.13289

**Published:** 2020-12-18

**Authors:** Qi Shen, Lei Liu, Xiaotong Gu, Da Xing

**Affiliations:** ^1^ MOE Key Laboratory of Laser Life Science & Institute of Laser Life Science South China Normal University Guangzhou China; ^2^ College of Biophotonics South China Normal University Guangzhou China

**Keywords:** Alzheimer's disease, AMPA receptor endocytosis, JNK3, Photobiomodulation therapy, synaptic dysfunction

## Abstract

Alzheimer's disease (AD), a severe age‐related neurodegenerative disorder, lacks effective therapeutic methods at present. Physical approaches such as gamma frequency light flicker that can effectively reduce amyloid load have been reported recently. Our previous research showed that a physical method named photobiomodulation (PBM) therapy rescues Aβ‐induced dendritic atrophy *in vitro*. However, it remains to be further investigated the mechanism by which PBM affects AD‐related multiple pathological features to improve learning and memory deficits. Here, we found that PBM attenuated Aβ‐induced synaptic dysfunction and neuronal death through MKP7‐dependent suppression of JNK3, a brain‐specific JNK isoform related to neurodegeneration. The results showed PBM‐attenuated amyloid load, AMPA receptor endocytosis, dendrite injury, and inflammatory responses, thereby rescuing memory deficits in APP/PS1 mice. We noted JNK3 phosphorylation was dramatically decreased after PBM treatment *in vivo* and *in vitro*. Mechanistically, PBM activated ERK, which subsequently phosphorylated and stabilized MKP7, resulting in JNK3 inactivation. Furthermore, activation of ERK/MKP7 signaling by PBM increased the level of AMPA receptor subunit GluR 1 phosphorylation and attenuated AMPA receptor endocytosis in an AD pathological model. Collectively, these data demonstrated that PBM has potential therapeutic value in reducing multiple pathological features associated with AD, which is achieved by regulating JNK3, thus providing a noninvasive, and drug‐free therapeutic strategy to impede AD progression.

## Graphical abstract

Inhibition of JNK3 phosphorylation by PBM treatment is powerfully effective in attenuating AMPA receptor endocytosis and reducing multiple neuropathologies associated with Alzheimer’s disease (AD), suggesting that PBM has potential therapeutic value in impeding AD progression, which is likely achieved by regulating JNK3.

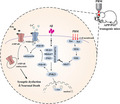

## 1 INTRODUCTION

In an aging population, Alzheimer's disease (AD) is one of the most common neurodegenerative disorders, characterized by the accumulation of amyloid‐β (Aβ) plaques and neurofibrillary tangles, synaptic and neuronal loss, and cognitive decline. One of the earliest signs of AD is the loss of synapses, which is at least partially linked to the toxicity mediated by Aβ, a peptide that accumulates in the brains of AD patients (Leshchyns'ka *et al*., 2015). Although the role of Aβ in AD pathogenesis remains controversial, Aβ clearly correlates with neuronal death and dendrite damage, and that may be causative in AD pathogenesis. The reduction in synapse numbers is the best neuropathological correlate to the degree of dementia in AD. Accumulating evidence indicates that transgenically produced Aβ or treatment with Aβ oligomers decreases dendritic spine density, impairs long‐term potentiation (LTP), and facilitates long‐term depression (LTD) (Birnbaum et al., 2015).

Photobiomodulation (PBM) therapy is a drug‐free noninvasive physical strategy with light spectrum from the visible to near‐infrared range that has been applied in dermatology, dentistry, immunology (Sato et al., 2016), neurology (De et al., 2011), and regenerative medicine. It is a nonthermal process involving endogenous chromophores eliciting photophysical and photochemical events at various biological scales (Anders et al., 2015). Recent study showed that noninvasive whole body illumination with PBM increased platelet generation and greatly ameliorated thrombocytopenia in mice (Zhang et al., 2016). Arany and colleagues found that PBM also known as low power laser treatment activates latent transforming growth factor‐β (TGF‐β), leading to the differentiation of dental stem cells and the formation of tertiary dentin (Arany et al., 2014). Recently, Tsai and colleagues showed that gamma frequency entrainment (40 Hz), a new noninvasive physical therapy, reduces amyloid levels and tau hyperphosphorylation (Iaccarino et al., 2016). These results suggest that drug‐free physical treatment, which is fundamentally different from previous AD therapies, represents a new approach for the treatment of AD.

A major obstacle to presymptomatic diagnosis and disease‐modifying therapy for AD is inadequate understanding of the molecular mechanisms of AD pathogenesis. This study aims to treat the disease by targeting molecular pathways that are responsible for the pathogenesis of AD through photobiomodulation. Previous studies have shown that the c‐Jun N‐terminal kinase (JNK) proteins, especially JNK3, a brain‐specific JNK isoform, potentially link together the major pathological hallmarks of AD (Hollos et al., 2020; Sato et al., 2002; Sclip et al., 2014; Sherrin et al., 2010). Deleting *JNK3* also results in a significant increase in neuronal and oligodendrocyte survival after traumatic injuries in the CNS (Li et al., 2007). It has been suggested that inhibition of JNK3 might have therapeutic utility in the treatment of AD. Mitogen‐activated protein kinase phosphatase 7 (MKP7), a JNK‐specific phosphatase, inactivates the region of β‐arrestin 2 combined with JNK3 (Masuda et al., 2003). Previous observations have suggested that activation of extracellular regulated protein kinase (ERK) induces phosphorylation of MKP7, therefore binding with scaffold proteins and inhibiting activation of JNK3 (Katagiri et al., 2005). However, whether activated ERK induced by PBM phosphorylates MKP7 is unclear. The most sensitive and typical expression of Aβ in the transmission of synaptic signaling is that LTP is inhibited (Jo et al., 2011) and LTD is abnormally enhanced (Li et al., 2009). Aβ induces the activation of calcineurin and the dephosphorylation of AMPA receptors (AMPARs), which reduces the postsynaptic number of membrane receptors; thus, the presynaptic release of neurotransmitter loses its sensitivity, causing the formation of LTD and eventually leading to synaptic dysfunction. Previous evidence has indicated that JNK promotes postsynaptic density‐95 (PSD‐95) phosphorylation, depressing the starting level of AMPA receptor endocytosis. We therefore hypothesized that PBM can inhibit AMPA receptor endocytosis by regulating JNK3, thus alleviating the synaptic dysfunction induced by Aβ.

In this study, we found that JNK3 activity is decreased in APP/PS1 transgenic mice via PBM (635 nm, 6 J/cm^2^, daily for 30 days), resulting in a dramatic reduction in amyloid load, AMPA receptor endocytosis, and inflammatory responses, thereby rescuing memory deficits. In AD experimental models, PBM activated ERK and subsequently phosphorylated and stabilized MKP7, resulting in JNK3 inactivation. We also found that PBM rescued not only the decrease of dendritic numbers and spine density, but also endocytosis and dephosphorylation of synaptic AMPA receptors. Collectively, our data demonstrate for the first time that inhibition of JNK3 phosphorylation by PBM treatment is powerfully effective in attenuating synaptic dysfunction and reducing multiple neuropathologies associated with AD, suggesting that PBM has potential therapeutic value in impeding AD progression, which is likely achieved by regulating JNK3.

## 2 RESULTS

### 2.1 Effects of PBM on neuronal damage in cortex and hippocampal regions of APP/PS1 transgenic mice

**Figure 1:**
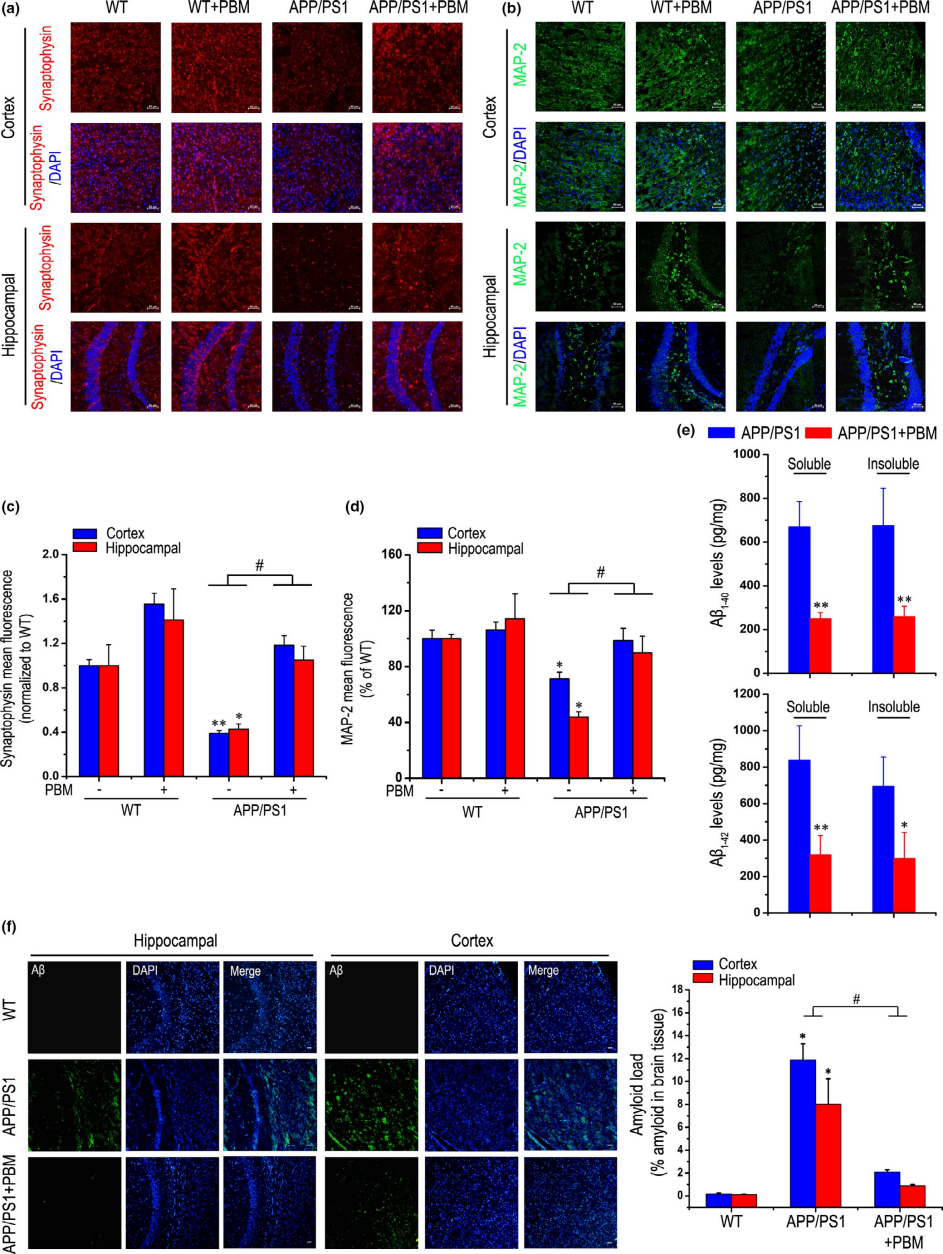
Effects of PBM on neuronal damage in cortex and hippocampus of APP/PS1 transgenic mice. (a) Representative images of synaptophysin (red) in hippocampal and cortex regions of each group; and DAPI labeling of cell nuclei (blue). Scale bar represents 50 μm. (b) Typical staining of MAP‐2 (green) in cortex and hippocampal regions from APP/PS1 or WT groups with or without PBM. Nuclei were counterstained with DAPI (blue). Scale bar: 50 μm. (c) Quantitative analyses of the synaptophysin mean fluorescence in the hippocampal and cortex regions of different group, respectively. The synaptophysin mean fluorescence was analyzed by ImageJ (*n* = 6 for each group, at least three individual experiments, mean ±SEM, two‐way ANOVA, *p < 0.05 vs. WT group; **p < 0.01 vs. WT group; #*p < *0.05 vs. indicated group). (d) Quantification of MAP‐2 density in hippocampal and cortex regions of different groups. MAP‐2 mean fluorescence was analyzed by ImageJ software (*n* = 6 for each group, at least three individual experiments, mean ±SEM, two‐way ANOVA, *p < 0.05 vs. WT group; #*p < *0.05 vs. indicated group). (e) Soluble and insoluble Aβ_1‐40_/Aβ_1‐42_ levels in APP/PS1 group with or without PBM. The Aβ measurements were performed by ELISA (*n* = 4 for each group, at least three individual experiments, mean ± SEM, Student's *t* test, *p < 0.05 vs. control transgenic group; **p < 0.01 vs. control transgenic group). (f) Histochemical and quantitative analyses of Aβ levels in the cortex and hippocampal regions of each group (*n* = 4–5 for each group, at least three individual experiments, mean ± SEM, two‐way ANOVA, *p < 0.05 vs. WT group; #*p < *0.05 vs. indicated group). Nuclei were counterstained with DAPI (blue). Scale bar, 50 μm. See Figure S1 for effects of PBM on Aβ load and neuroinflammation in APP/PS1 mice

The APP/PS1 model is known to develop AD‐like phenotypes from 3 months of age (Kim et al., 2015). In order to evaluate the validity of PBM treatment, brain sections were stained with synaptic marker synaptophysin, which are involved synaptic transmission, and learning and memory. As shown in Figure 1a and c, the synaptophysin fluorescent staining intensity of hippocampal and cortex regions in the PBM‐treated group was higher than in the non‐PBM‐treated group. More importantly, there was significant, reduced synaptophysin density in the cortex of APP/PS1 mice compared with wild‐type (WT) mice, but PBM remarkably ameliorated it. These results suggest that PBM regulates synaptic proteins, leading us to speculate that PBM might also modulate synaptic connectivity and dendritic morphology. To further investigate the effects of PBM on Aβ‐induced dendrite damage, brain sections were stained with MAP‐2 (microtubule associated protein‐2, MAP‐2), a sensitive indicator for the assessment of neuronal injury. Compared with WT mice, APP/PS1 mice had less MAP‐2 fluorescent staining intensity both in the cortex and hippocampal region (Figure 1b and d). In contrast, the MAP‐2 fluorescent staining intensity was increased significantly in PBM‐treated APP/PS1 mice. Enzyme‐linked immunosorbent assay (ELISA) for Aβ revealed a dramatic reduction in soluble Aβ_1‐40_ and Aβ_1‐42_, even in insoluble Aβ_1‐40_ and Aβ_1‐42_, in APP/PS1 mice after treated with PBM (Figure 1e). Furthermore, we also analyzed the effect of PBM on overall Aβ depositions (Figure 1f, and Figure S1b); the area of amyloid‐β was strongly reduced in the cortex and hippocampus isolated from PBM‐treated AD mice compared with the APP/PS1 group. Taken together, PBM attenuated dendrite injury, amyloid load, and neuroinflammation (Figure S1c–f), suggesting that PBM may be effective at impeding AD progression.

### 2.2 Effects of PBM on memory impairment of APP/PS1 transgenic mice

**Figure 2:**
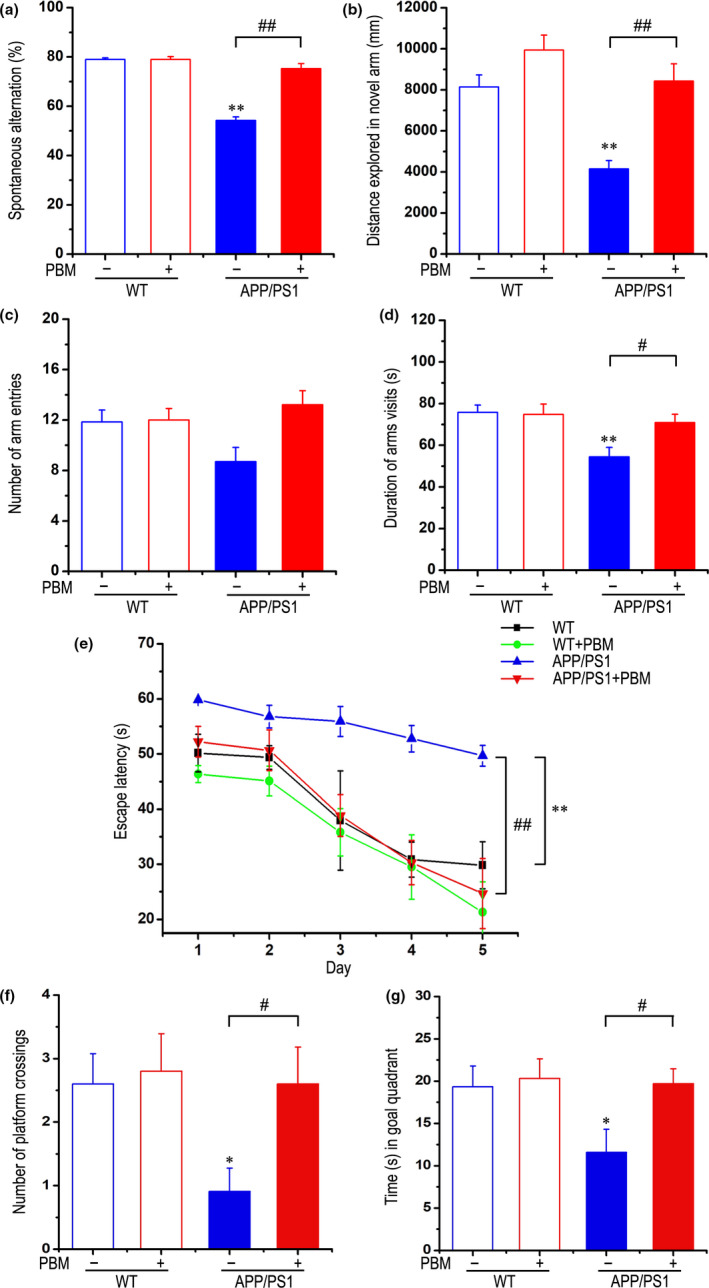
Effects of PBM on memory impairment of APP/PS1 transgenic mice. Y‐maze task: (a) Spontaneous alternation behavior was measured in APP/PS1 or WT groups with or without PBM. (b–d) The distance explored (b), duration time (d) in novel arm, and number of arm entries (c) of each group were measured. Morris water maze task: (e) The escape latency of mice to find the hidden platform was recorded on each training trial day. (f) Number of platform crossings during a 60 s probe trial of MWM test. (g) Time spent swimming in the goal quadrant during the probe trial. All data are presented as mean ± SEM from 12–14 mice in each group. *p < 0.05 vs. WT group, **p < 0.01 vs. WT group, #*p < *0.05 vs. indicated group, ##*p < *0.01 vs. indicated group by two‐way ANOVA. See Figure S2 for effects of PBM on the average speed in the Y‐maze task and the Morris water maze

To test whether PBM protection against Aβ neurotoxicity extended to memory, we assessed PBM‐treated and non‐PBM‐treated APP/PS1 group as well as WT group with or without PBM treated on tests of spatial memory. Spontaneous alternation, which is regarded as a measure of spatial memory, was investigated using the Y‐maze test. The APP/PS1 transgenic mice showed impaired behaviors, but interestingly, PBM treatment significantly rescued the deficits of spontaneous alternation behavior (Figure 2a). As shown in Figure 2b and d, distance explored and the total duration of visits in the novel arm were significantly increased in PBM‐treated group compared with the non‐PBM‐treated AD mice model group. However, as shown in Figure 2c, total number of arm entries which is regarded as locomotor activity was not significantly different among groups. Furthermore, there was no significant difference in average speed among groups (Figure S2a,b).

We next applied the Morris water maze (MWM) test to evaluate spatial learning and memory for the location of hidden platform relative to surrounding cues (Vorhees & Williams, 2006). All groups identified hidden platforms in successive training trials, although the escape latency of PBM‐treated group was significantly shorter than non‐PBM‐treated AD mice group (Figure 2e). However, the average swimming speed did not differ significantly among the groups in this trial (Figure S2c). During the probe trial, APP/PS1 mice that received PBM treatment spent a significantly longer time exploring the goal quadrant and had a higher number of crossings over the platform, versus nontreated APP/PS1 mice (Figure 2f,g). There was no significant difference in average swimming speed among groups (Figure S2d). Together, these results show that PBM treatment can effectively improve spatial learning and memory in APP/PS1 mice.

### 2.3 PBM inhibits JNK3 phosphorylation in APP/PS1 transgenic mice

**Figure 3:**
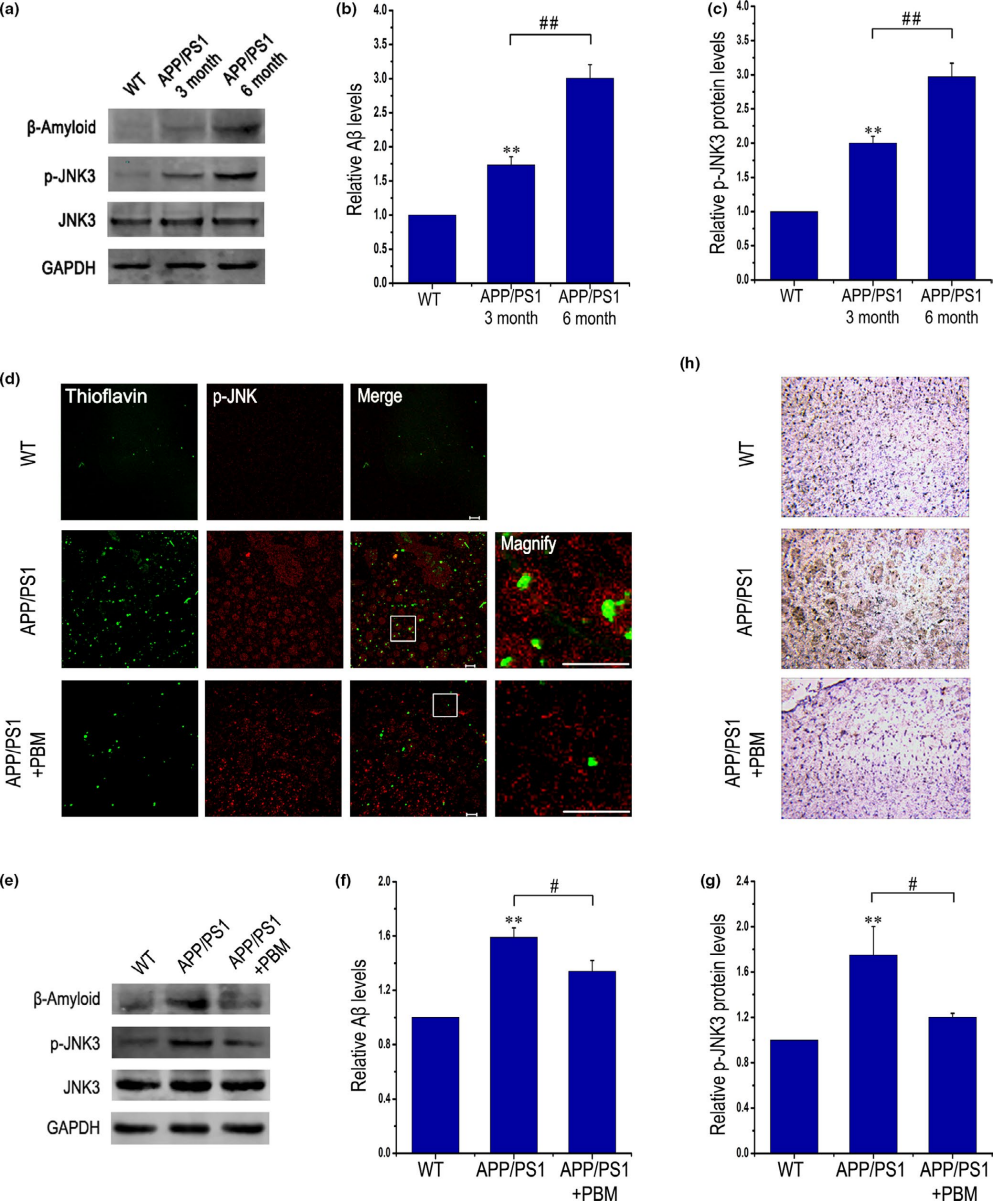
PBM inhibits JNK3 phosphorylation in APP/PS1 transgenic mice. (a–c) Western blot analysis of cortex lysates from WT mice and APP/PS1 transgenic mice at three and 6 months of age (*n* = 3 for each group, at least three individual experiments, mean ± SEM, one‐way ANOVA, **p < 0.01 vs. age‐matched WT group, ##*p < *0.01 vs. indicated group). (d) Representative immunofluorescent images of p‐JNK in the cortex. Also shown are images of thioflavin T staining from the frontal cortex. Scale bar, 20 μm. (e–g) Aβ and p‐JNK expression were detected by Western blot in cortex lysates from APP/PS1 transgenic mice with or without PBM (6 J/cm^2^) and age‐matched WT mice (*n* = 5, at least three individual experiments, mean ± SEM, two‐way ANOVA, *p < 0.05 vs. control group, **p < 0.01 vs. control group, #*p < *0.05 vs. indicated group, ##*p < *0.01 vs. indicated group). (h) Immunohistochemical test performed to detect p‐JNK in sections of brain from APP/PS1 transgenic mice with or without PBM (6 J/cm^2^) and age‐matched WT mice; original magnification of 20 times. See Figure S3 for inhibition of PBM on JNK3 phosphorylation and APP Thr668 phosphorylation in an AD transgenic mouse model. And also please refer to Figure S4 to view that the potential disease‐modifying therapeutic mechanism of PBM on memory impairment and Aβ load, which is likely achieved by regulate JNK3, in APP/PS1 mice

A growing body of evidence indicates that JNK3 activity is increased in human AD brain (Yoon et al., 2012). We first characterized changes of p‐JNK3 levels in the APP/PS1 transgenic mice model at 3 and 6 months of age (Figure 3a). Compared with 3‐month‐old APP/PS1 brains, we observed a significant increase in Aβ levels throughout the entire cerebral cortex of 6‐month‐old APP/PS1 mice (Figure 3a,b). Indeed, JNK3 activity increased in 3‐month‐old APP/PS1 mice compared with age‐matched WT mice, and the increase was age‐dependent (Figure 3a,c).

We next determined the effect of PBM (6 J/cm^2^) on the amount of Aβ plaques in the brain and the location of active JNK in APP/PS1 brains using p‐JNK antibody. Beginning at 6 months, the time when plaques appear in APP/PS1 mice, p‐JNK signals were predominantly detected near plaque structures, colocalizing with thioflavin T (Figure 3d). In contrast, p‐JNK signals were rarely detected around plaques in APP/PS1 mice treated with PBM (Figure 3d). Importantly, PBM effectively reduced the level of p‐JNK3 compared with non‐PBM‐treated APP/PS1 mice (Figure 3e–h). These results strongly indicated that JNK3 activity and Aβ deposits are decreased in transgenic mice via PBM treatment.

We further provide a direct approach that use of SP600125 (JNK3 inhibitor, 30 mg/kg, i.p) in conjunction with PBM or not, to show whether the effect mediated by PBM is mainly via inhibition of JNK3. As shown in Figure S4a–e, the Y‐maze test was performed to test spatial memory. Compared with APP/PS1 group, PBM‐treated APP/PS1 group significantly rescued the deficits of spontaneous alternation behavior (Figure S4a). In addition, whether SP60012‐treated APP/PS1 group or SP600125 combined with PBM‐treated APP/PS1 group significantly alleviated the defects of spontaneous alternation behavior in APP/PS1 mice, but there was no significant difference between the SP600125‐treated APP/PS1 group and the SP600125 combined with PBM‐treated APP/PS1 group. As shown in Figure S4b and d, distance explored and the total duration of visits in novel arm were significantly increased in PBM‐treated APP/PS1 group compared with APP/PS1 group. SP600125 combined with PBM‐treated APP/PS1 group showed similar results. But SP600125‐treated APP/PS1 group showed no significant difference with APP/PS1 group in the total duration of visits in novel arm (Figure S4d). Furthermore, ELISA for Aβ revealed a dramatic reduction in soluble Aβ_1‐40_ and Aβ_1‐42_, even in insoluble Aβ_1‐40_ and Aβ_1‐42_ in APP/PS1 mice after treated with PBM (Figure S4f). Similar results were obtained in APP/PS1 treated with SP600125 and PBM group. But compared with SP600125 combined with PBM treatment group in APP/PS1, a less reduction in soluble Aβ_1‐40_ and Aβ_1‐42_, even in insoluble Aβ_1‐40_ and Aβ_1‐42_ in APP/PS1 mice after treated with SP600125. We next tested p‐JNK3 and p‐c‐Jun levels *in vivo* by Western blotting (Figure S4g–h). Quantitative analysis revealed that the levels of p‐JNK3 and p‐c‐Jun were markedly decreased in the cerebral cortex in PBM‐treated and PBM combined with SP600125‐treated APP/PS1 mice compared with APP/PS1 mice (*p* < 0.01), whereas SP600125‐treated APP/PS1 mice resulted in a less decrease of p‐JNK3 and p‐c‐Jun in cerebral cortex (*p* < 0.05). Taken together, it is suggested that although the effect mediated by PBM is mainly achieved by inhibiting JNK3 in APP/PS1 mice, PBM may have a pleiotropic effect.

### 2.4 Activated ERK induced by PBM inactivates JNK3 under treatment with Aβ

**Figure 4:**
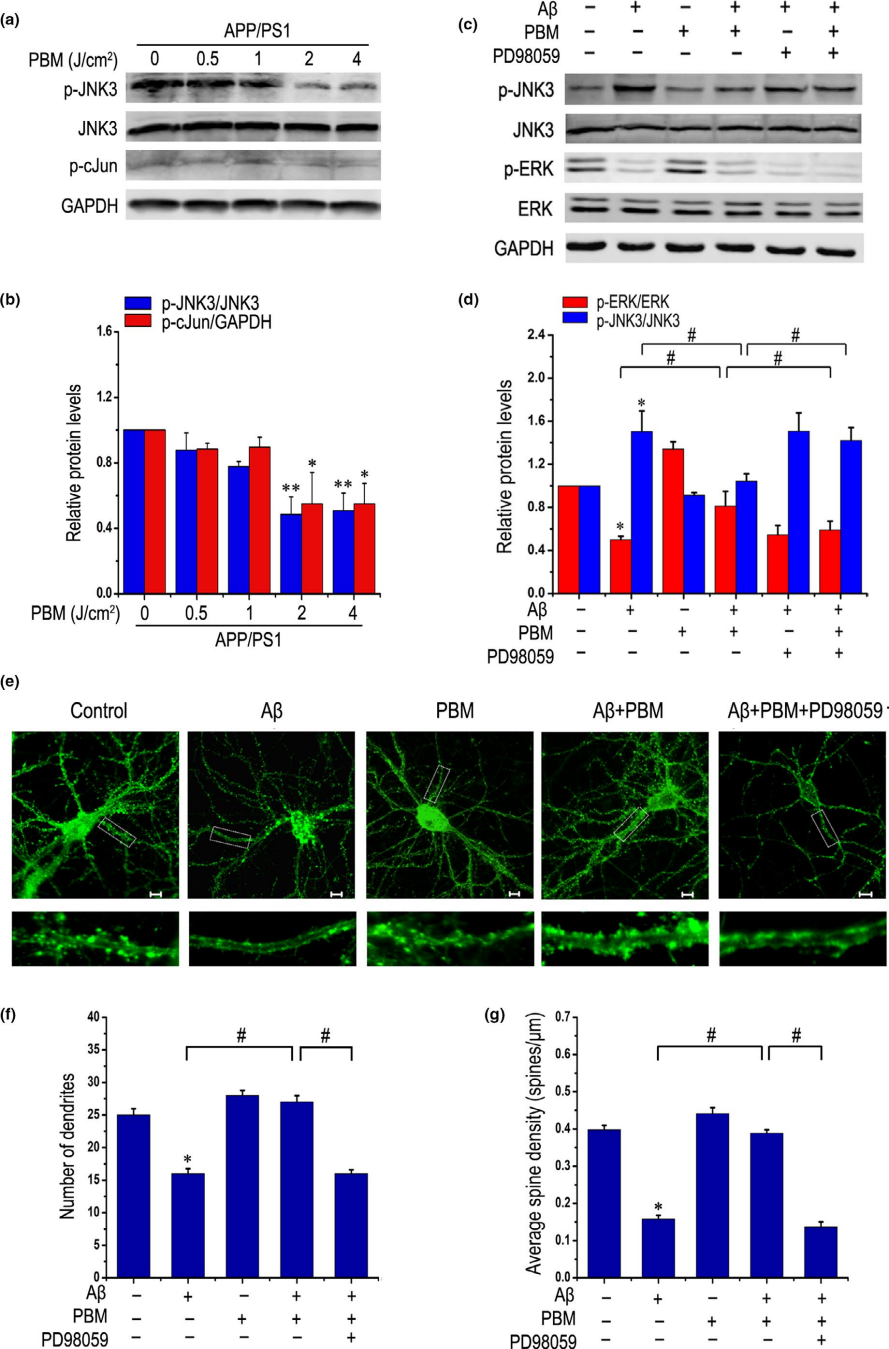
Activated ERK induced by PBM inactivates JNK3 under treatment with Aβ. (a, b) Representative Western blot assay for detecting dose response of PBM (0.5, 1, 2, 4 J/cm^2^) on p‐JNK3, JNK3, and p‐c‐Jun levels in primary neurons derived from APP/PS1 mice (at least three individual experiments, mean ± SEM, one‐way ANOVA, *p < 0.05 vs. control group; **p < 0.01 vs. control group). (c, d) Representative Western blot assay of p‐ERK, ERK, p‐JNK3, and JNK3 stimulated with Aβ_1‐42_ and/or PBM in the presence of PD98059 (1 μM) in primary neurons (at least three individual experiments, mean ± SEM, one‐way ANOVA, *p < 0.05 vs. control group; #*p < *0.05 vs. indicated group). (e) Representative photomicrographs of FITC‐phalloidin labeling in primary neurons on 14 DIV under treatment with Aβ_1‐42_ and/or PBM in the presence of PD98059. Scale bar: 10 μm. (f) Quantification of the number of dendrites per neuron under indicated treatments. For each group, >20 neurons were measured. (g) Quantification of spine density under indicated treatments. For each group, we measured >20 dendrites. All data are presented as means ± SEM for at least three individual experiments. *p < 0.05 vs. control group, #*p < *0.05 vs. indicated group by one‐way ANOVA procedure. Please refer to Figure S5 to view that PBM inhibits phosphorylated form of JNK3 in Aβ‐treated primary neurons through ERK‐mediated signaling pathway. And also refer to Figure S7 to view the effect of PBM on dendritic spine density of APP/PS1 mice by Golgi‐Cox staining

Previous studies have shown that JNK3 plays a critical role in the process of Aβ‐induced neuronal apoptosis (Li et al., 2007). To examine the optimal dose of PBM, Aβ_1‐42_ treated primary neurons or APP/PS1 primary neurons were exposed to different dose of PBM (0.5, 1, 2, 4 J/cm^2^). As shown in Figure S5a and b, a decrease in p‐JNK3 as well as in p‐c‐Jun were seen in the samples after PBM treatment at the dose of 2 and 4 J/cm^2^, even in the Aβ_1‐42_‐treated group. Similar results were obtained in APP/PS1 primary neurons (Figure 4a,b). And combined with previous published results, 2 J/cm^2^ was chosen in as the optimum irradiation dose in this study (Meng et al., 2013). Thus, JNK3 was phosphorylated in response to Aβ, whereas PBM abolished that effect.

We next tested specific inhibitors of these pathways: API‐2 and PD98059 for Akt, and MEK/ERK, respectively, by Western blotting (Figure S5c,d). We demonstrated that PBM inhibits the phosphorylation of JNK3, which is reversed by PD98059, but not API‐2, suggesting that MEK/ERK signaling is obligatory for PBM‐stimulated JNK3 dephosphorylation in Aβ‐treated neurons. As shown in Figure 4c and d, a decrease in p‐JNK3 and an increase in p‐ERK were seen in the samples after PBM treatment, even in the Aβ_1‐42_‐treated group. PD98059 reduced the level of p‐ERK induced by PBM and also blocked the suppression of PBM to JNK3. Taken together, these results demonstrated that activated ERK induced by PBM subsequently inactivated JNK3 under the treatment with Aβ. Moreover, CCK‐8 assay and flow cytometric analysis were performed to detect cell viability and apoptosis (Figure S6a–f). Compared with the Aβ_1‐42_‐treated group, relative cell viability was increased after treatment with PBM. The ERK inhibitor PD98059 negated PBM protection against neurotoxicity. These results further confirmed that PBM attenuated Aβ_1‐42_‐induced neuronal neurotoxicity, which was dependent on ERK.

Before Aβ‐induced neuronal apoptosis, dendritic spine loss and synaptic dysfunctions already exist. Phalloidin staining which were performed to examine morphological changes of dendrites showed that dendritic atrophy of primary APP/PS1 neurons was rescued by PBM (Figure S6g,h), which are directly related to memory loss. To further determine the effect of PBM on dendritic spines *in vivo*, we quantified dendritic spines using Golgi‐Cox staining, a method used for counting dendritic spines (Manczak et al., 2018), in WT and APP/PS1 mice treated with or without PBM. As shown in Figure S7, we found significantly reduced spines in APP/PS1 mice relative to WT mice, but PBM rescued the decreased of dendritic spines. We next determined whether inactivated JNK3 by PBM via ERK signaling regulates dendrite patterning in Aβ_1‐42_‐treated neurons. PBM significantly increased the dendrite numbers and spine density of Aβ_1‐42_‐treated primary neurons. However, inhibition of ERK reversed these results (Figure 4e–g). In addition, a similar result was obtained in Figure S6i by using another method of cellular immunofluorescence of MAP‐2, which is also recognized for morphological characterization of dendrites. These results revealed that PBM activated ERK to inhibit the phosphorylation of JNK3 and rescued dendritic damage.

### 2.5 ERK activated by PBM promotes MKP7 phosphorylation, and MKP7 interacts with and inactivates JNK3

**Figure 5:**
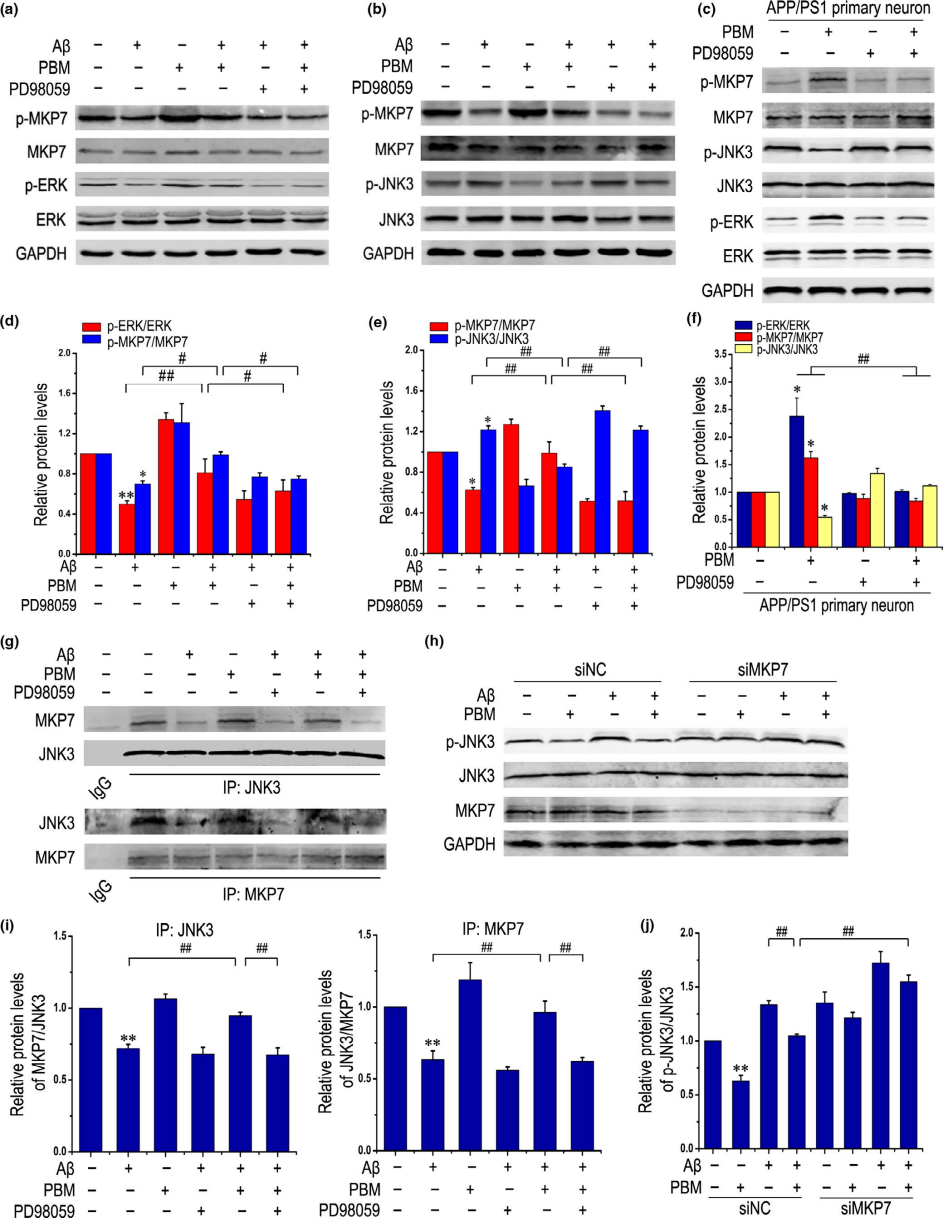
ERK activated by PBM promotes MKP7 phosphorylation, and MKP7 interacts with and inactivates JNK3. (a, d) Primary neurons were treated with Aβ_1‐42_ and/or PBM, or pretreated with PD98059. Representative Western blots for detecting the levels of p‐MKP7, total‐MKP7, p‐ERK, and ERK are shown. All data are presented as means ± SEM for at least three individual experiments. *p < 0.05 vs. control group, **p < 0.01 vs. control group, #*p < *0.05 vs. indicated group, ##*p < *0.01 vs. indicated group by one‐way ANOVA. (b, e) Representative Western blot analysis was performed to detect p‐MKP7, MKP7, p‐JNK3, JNK3 stimulated with Aβ_1‐42_ and/or PBM in the presence of PD98059 in primary neurons (at least three individual experiments, mean ±SEM, one‐way ANOVA, *p < 0.05 vs. control group, ##*p < *0.01 vs. indicated group). (c, f) p‐MKP7, MKP7, p‐JNK3, JNK3, p‐ERK, and ERK protein levels were detected by Western blots in primary neurons derived from APP/PS1 mice on 14 DIV under the indicated treatments (at least three individual experiments, mean ± SEM, one‐way ANOVA, *p < 0.05 vs. APP/PS1 group, ##*p < *0.01 vs. indicated group). (g, i) Representative Western blots showing co‐immunoprecipitation of JNK3 with MKP7 from primary neurons stimulated with Aβ_1‐42_ and/or PBM in the presence of PD98059 (at least three individual experiments, mean ± SEM, one‐way ANOVA, **p < 0.01 vs. control group, ##*p < *0.01 vs. indicated group). (h, j) Representative Western blot analysis of p‐JNK3, JNK3, and MKP7 under the indicated treatments (at least three individual experiments, mean ± SEM, two‐way ANOVA, **p < 0.01 vs. control group, ##*p < *0.01 vs. indicated group). See Figure S9 for siRNAs used in this study

Since MKP7 has potential links to ERK and JNK3 (Masuda et al., 2001), we tested whether PBM exerted its neuroprotective effects by activating ERK and, as a result, promoting MKP7 phosphorylation. Data shown in Figure 5a and d revealed that PBM alone activated ERK and MKP7, and effectively reversed the decrease in phosphorylation levels of ERK and MKP7 in primary neurons exposed to Aβ_1‐42_. PD98059 inhibited the activation of ERK and abolished the activating effect of PBM on MKP7.

The dual‐specificity protein phosphatase MKP7, a negative regulator of the MAPK signaling pathway, specifically inactivates JNK signaling (Lee et al., 2010). We further investigated the effect of MKP7 on the phosphorylation of JNK3 in response to PBM in the Aβ_1‐42_‐treated group. The results showed a significant increase of MKP7 phosphorylation, but a lower level of JNK3 phosphorylation in the PBM‐treated group, even in the presence of Aβ_1‐42_. However, PD98059 reversed the effect of PBM (Figure 5b and e). Similar results were obtained in APP/PS1 primary neurons (Figure 5c and f).

We next tested the mechanism by which PBM inhibited JNK3 activity, using a co‐IP experiment to explore the interaction between MKP7 and JNK3 in primary neurons. As shown in Figure 5g and i, compared with the untreated group, the amount of MKP7 binding to JNK3 (refer to the protein levels of MKP7/JNK3) was obviously decreased in the Aβ_1‐42_‐treated group. Conversely, the amount increased significantly in response to PBM, indicating a stronger interaction between MKP7 and JNK3. When PD98059 was added, the amount was reversed. Similar results were observed in the amount of JNK3 binding to MKP7 (refer to the protein levels of JNK3/MKP7). Furthermore, knocking down MKP7 abolished the suppression of JNK3 activation by PBM (Figure 5h,j and Figure S8). Altogether, JNK3 was activated in response to Aβ_1‐42_, whereas PBM inactivated it in an MKP7‐dependent manner.

### 2.6 PBM inhibits phosphorylation of PSD‐95 and AMPA receptor endocytosis, thereby alleviating synaptic dysfunction

**Figure 6:**
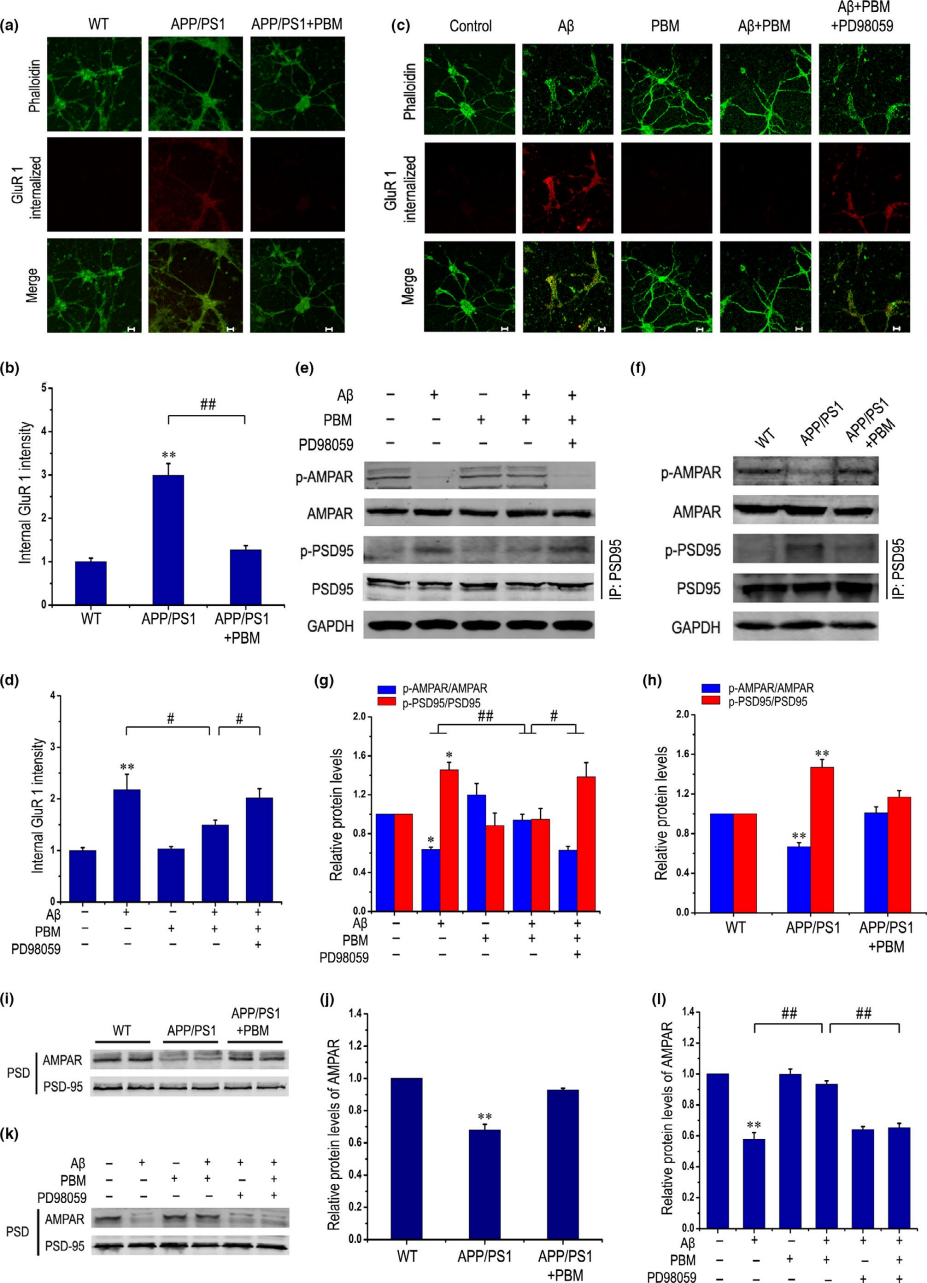
PBM inhibits phosphorylation of PSD‐95 and AMPA receptor endocytosis, thereby alleviating synaptic dysfunction. (a, b) Representative images (a) and quantitation (b) of total endocytosis of AMPARs (GluR1 internalized) in primary neurons derived from APP/PS1 mice under treatment with or without PBM (five individual experiments, mean ± SEM, two‐way ANOVA, **p < 0.01 vs. control group, ##*p < *0.01 vs. indicated group). Scale bar: 20 μm. (c) Representative images of total endocytosis of AMPARs (GluR1 internalized) in response to different treatments. Scale bar: 20 μm. (d) Quantitation of total endocytosis of AMPARs stimulated with various treatments in primary neurons (at least three individual experiments, mean ± SEM, one‐way ANOVA, **p < 0.01 vs. control group, #*p < *0.05 vs. indicated group). (e, g) Representative Western blot analysis was performed to detect p‐AMPAR, and AMPAR stimulated with Aβ_1‐42_ and/or PBM in the presence of PD98059 in primary neurons. Immunoprecipitates were analyzed for detecting p‐PSD95, and PSD95 stimulated with various treatments in primary neurons (at least three individual experiments, mean ± SEM, one‐way ANOVA, *p < 0.05 vs. control group, #*p < *0.05 vs. indicated group, ##*p < *0.01 vs. indicated group). (f, h) Representative Western blot assay of p‐AMPAR, and AMPAR, and immunoprecipitation of p‐PSD95 and PSD95 in neurons derived from APP/PS1 mice under treatment with or without PBM (at least three individual experiments, mean ± SEM, two‐way ANOVA, **p < 0.01 vs. control group). (i, j) Representative immunoblots of PSD proteins from APP/PS1 mice treated with or without PBM (at least three individual experiments, mean ± SEM, two‐way ANOVA, **p < 0.01 vs. control group). (k) Representative Western blots of PSD proteins from cultured neurons in response to different treatments. (l) Quantification of (k). Data are presented as means ± SEM for at least three individual experiments. **p < 0.01 vs. control group; ##*p < *0.01 vs. indicated group. Please refer to Figure S10 to see that inhibition of JNK3 can alleviate AMPA receptor endocytosis

Aβ accumulation in the brain is associated with or possibly induces the dysfunction of synapses and memory impairments, contributing to the pathogenesis of AD. Therefore, we determined whether PBM rescued hippocampal synaptic impairment in cultured APP/PS1 primary neurons. Consistent with previous results using a method that allows visualization of internalized AMPARs (Bhattacharyya et al., 2009), PBM treatment prevented the endocytosis of AMPARs in APP/PS1‐cultured neurons (Figure 6a,b). Because inhibition of JNK3 activity by PBM established a neuroprotective effect in APP/PS1 mice (Figures 1 and 2) and Aβ‐treated primary neurons (Figure 4), we determined whether inhibition of JNK3 activation is sufficient to rescue synapse impairment caused by endocytosis of AMPARs. Aβ_1‐42_ treatment led to the endocytosis of surface AMPARs, and co‐treatment with PBM or SP600125 (a JNK inhibitor) abolished the endocytosis (Figure S9b,c). Moreover, PBM inhibited JNK3 activation through the ERK/MKP7 pathway in neurons even when exposed to Aβ_1‐42_ (Figures 4 and 5). As shown in Figure 6c and d, PBM significantly reduced the levels of AMPAR internalization even in the Aβ_1‐42_‐treated group, but PD98059 removed the effect. Additional data showed that PBM significantly increased surface AMPAR levels even in neurons treated with Aβ_1‐42_, but PD98059 abolished it (Figure S9a). In addition, Western blotting was performed to quantify the phosphorylation of AMPA receptor GluR 1 subunit, which indicated AMPA receptor on the surface in cultured neurons. PBM increased AMPAR phosphorylation even in neurons treated with Aβ_1‐42_ or derived from APP/PS1 mice, suggesting that PBM as a noninvasive physical treatment markedly rescued Aβ‐induced AMPA receptor endocytosis (Figure 6e–h).

PSD‐95 is an abundant scaffold protein in the postsynaptic density of excitatory synapses that exerts a strong positive influence on synaptic strength (El‐Husseini et al., 2000). PSD‐95 is involved in synaptic AMPAR endocytosis, which is a mediators of the vast majority of excitatory synaptic transmissions in the CNS (Qin et al., 2015). Thus, we determined whether PBM inhibits PSD‐95 phosphorylation in neurons exposed to Aβ_1‐42_. As shown in Figure 6e and g, PBM markedly inhibited PSD‐95 phosphorylation in Aβ_1‐42_‐treated neurons. When PD98059 was added, the results were reversed, indicating that PBM inhibited phosphorylation of PSD‐95, and AMPA receptor endocytosis depended on ERK activation.

Underneath the postsynaptic plasma membranes resides a protein‐rich subcompartment known as PSD, which is a component that transmits presynaptic terminal signals (Zeng et al., 2016). As PSD modifications precede functional alterations of synapses, we analyzed proteins in hippocampal and cultured neuron PSD fractions. As expected, PBM restored AMPAR levels in APP/PS1 mice (Figure 6i and j). As shown in Figure 6k and l, PBM rescued the surface expression of the AMPAR GluR 1 subunit in Aβ_1‐42_‐treated neurons, but inhibiting ERK reversed the results. Furthermore, we used SP600125 (JNK3 inhibitor, 30 mg/kg, i.p) to inject APP/PS1 mice with or without PBM treatment for 30 days to illustrate whether PBM alleviates multiple pathological features associated with AD models by regulating JNK3. As shown in Figure S9d, we also detected the phosphorylation level of AMPA receptor subunit GluR1 by Western blotting experiment and analyzed proteins in PSD fractions. PBM increased the phosphorylation level of AMPA receptor subunit GluR1 and restored AMPAR levels in APP/PS1 mice. Similar results were obtained in SP60125 combined with PBM‐treated APP/PS1 group. But SP600125‐treated APP/PS1 group resulted in a less increase of the phosphorylation level of AMPA receptor subunit GluR1 and the AMPA receptor levels in PSD fractions compared with SP600125 combined with PBM‐treated APP/PS1 group. Collectively, activation of ERK/MKP7 signaling by PBM significantly inhibited JNK3 activation and rescued AMPA receptor endocytosis in an AD pathological model.

## 3 DISCUSSION

During AD pathogenesis, Aβ monomers aberrantly aggregate into toxic oligomers, fibrils, and eventually plaques (Antoniou et al., 2011). Excessive Aβ causes neuronal apoptosis, facilitating the occurrence and progress of AD. In addition to neuronal death, dendrite numbers and spine density are obviously decreased in APP/PS1 transgenic mice, which produce high levels of human Aβ_1‐40_ and Aβ_1‐42_. Aβ can induce neurodegeneration, at least in part, through the JNK3 pathway (Braithwaite et al., 2010), suggesting that inhibition of JNK3 activity may be of therapeutic utility in the treatment of AD (Resnick & Fennell, 2004). A recent study has shown that chronic treatment with SP600125, a small molecular JNK inhibitor, results in a marked improvement of cognitive ability and a dramatic reduction in plaque burden (Zhou et al., 2015). In our present study, we observed that JNK3 activity was increased in an APP/PS1 transgenic mouse model (Figure 3a) and in Aβ_1‐42_‐treated primary neurons (Figure 4c). Our data showed a pro‐survival effect of PBM on Aβ_1‐42_‐induced neurotoxicity (Figure S6), and we found, for the first time to our knowledge, that PBM inhibited JNK3 activity via the ERK/MKP7 pathway (Figure 5). Additionally, PBM, which is a novel and drug‐free photobiomodulation method, attenuated dendritic injury, synaptic dysfunction, amyloid load (Figure 1 and Figure S1b), memory impairment (Figure 2), and neuroinflammation (Figure S1c–f) in APP/PS1 transgenic mice. These results suggest that photobiomodulation may potentially be used to treat AD by regulating JNK3.

Photobiomodulation therapy consists of nonthermal irradiation using light in the visible to near‐infrared range and has been used clinically to reduce pain and inflammation in a variety of pathologies (Schindl et al., 1999). In this study, ERK was phosphorylated by PBM even in the presence of Aβ_1‐42_ (Figure 4c). Activated ERK then subsequently phosphorylated MKP7 (Figure 5a) and stabilized MKP7 to perform its function (Figure 5g). MKP7 works as a JNK3‐specific phosphatase through binding with its scaffold proteins. Increasing evidence shows that MKP7 binds β‐arrestin 2 via amino acids 394–443 of MKP7, the same region that interacts with JNK‐interacting protein‐1 (JIP‐1) and inactivates the bound subset of JNK3 (Willoughby & Collins, 2005). It has been suggested that JIP‐1‐mediated JNK activation negatively regulates synaptic plasticity and spatial memory (Morel et al., 2018). In this study, we showed for the first time that PBM inhibited JNK3 activation through the ERK/MKP7 pathway in neurons even when exposed to Aβ_1‐42_.

In our current study, we confirmed that PBM rescued overall Aβ load, a classic hallmark of AD, in an APP/PS1 mouse model (Figure 1e,f and Figure S1b). A previous study showed that JNK3 promotes further APP processing by phosphorylating it at T668, generating, and accumulating more Aβ_1‐42_, thus exacerbating AD pathology (Mazzitelli et al., 2011). Furthermore, previous findings showed that active JNK is involved in the expression of BACE1 (Guglielmotto et al., 2011) and PS1 (Shen et al., 2008). These studies suggest that PBM results in Aβ reduction by inhibiting the amyloidogenic pathway and is a promising therapeutic strategy via inhibiting JNK3 (Figure S3).

Mounting evidence indicates that microglial activation in the central nervous system is heterogeneous, which can be categorized into two opposite types: M1 phenotype and M2 phenotype (Tang & Le, 2016). Microglia in this M1 phenotype act as neurotoxic cells that produce pro‐inflammatory cytokines (von Leden et al., 2013). In contrast, M2 microglia express the interleukin 4 receptor, mannose receptor, TGF‐β, and macrophage colony stimulating factor receptor (Michelucci et al., 2009). Recently, M1/M2 paradigm of microglial activation has been increasingly studied in several neurodegenerative diseases in attempt to uncover the mechanisms of immunopathogenesis. Our current study showed that PBM treatment effectively reduced neuroinflammation (Figure S1c–f) in APP/PS1 mice. And previous studies showed that PBM could decrease the level of reactive gliosis and pro‐inflammatory cytokine release induced by stroke (Yang et al., 2018). PBM could also attenuate pro‐inflammatory responses in microglia and enhance phagocytic activity through Src/PI3 K/Akt/Rac1 signaling pathway (Song et al., 2012). Mechanistic studies suggested beneficial effects of PBM were accompanied by the release of anti‐inflammatory cytokines, cytochrome c oxidase activity, and ATP production (Hamblin, 2017). Those results mentioned above indicated that the modulatory actions of PBM on AD, release of anti‐inflammatory cytokines, and transformation between the two phenotypes of activated microglia may contribute to decrease inflammatory damage and improve the neuronal microenvironment. There are two sides to everything. Similarly, we should not ignore the potential deleterious effects of PBM while elaborating that PBM has potential therapeutic value in impeding AD progression. Although the current experimental data show that transcranial PBM is safe and well tolerated, and there are no documents incidents of retinal or skin injury in patients treated with PBM, we should also pay attention to it. Given the potential risk of injury when high‐powered NIR devices are used by untrained personnel or laypersons, over‐the‐counter availability is not recommended for devices with high transmitting power (>1 W) (Cassano et al., 2016; Kim et al., 2007).

Alzheimer's disease is characterized by loss of synapses, which is the strongest correlate with cognitive decline, and possibly one of the earliest events in AD pathogenesis (Masliah et al., 2001). Clinical studies show that only targeting plaque load or neurofibrillary tangles induced by tau hyperphosphorylation cannot achieve successful treatment. Before the formation of amyloid plaques, neurons have already been functionally damaged; thus, alleviating neuronal synaptic dysfunction is a potential therapy. Here, we further found that PBM remarkably increased the dendritic spines in neurons derived from APP/PS1 transgenic mice (Figure S6g) and Aβ_1‐42_‐treated neurons (Figure 4e). AMPARs mediate the vast majority of excitatory neurotransmissions in the brain. Regulation of AMPARs plays critical roles in forms of synaptic plasticity such as LTP and LTD (Thomas et al., 2008). The downscaling of AMPARs occurs selectively in the cortex and CA1 of the hippocampus, regions that are most vulnerable to AD pathogenesis (Zhao et al., 2010). Immunofluorescence analysis showed that the number of GluR 1 clusters undergoing endocytosis was increased in neurons exposed to Aβ_1‐42_ compared with untreated group. However, PBM treatment significantly reduced the effect of Aβ_1‐42_ on AMPAR endocytosis (Figure 6c and d). The detailed molecular mechanisms underlying the trafficking of AMPARs into and out of synapses are of great interest because of their importance in prominent forms of synaptic and experience‐dependent plasticity (Bhattacharyya et al., 2009). PSD‐95 is an abundant postsynaptic scaffolding protein that is enriched in the PSD, regulating the formation, function, and plasticity of excitatory synapses (Han & Kim, 2008). Previous evidence has shown that biochemical modifications of PSD‐95 contribute to Aβ_1‐42_‐induced endocytosis in cultured neurons (Xu et al., 2008). JNK can promote PSD‐95 phosphorylation, stabilizing it at the spines, and depress the starting level of AMPAR endocytosis. In AD models, synaptic dysfunction induced by Aβ is dependent on NMDAR overstimulation (Knafo et al., 2016), leading to abnormal intracellular responses and excessive extracellular Ca^2+^ influx. Activation of NMDAR induces the dephosphorylation of AMPAR by calcineurin, which leads to AMPAR endocytosis (Jurado et al., 2010), and absence of AMPAR from the postsynaptic membrane, resulting in synaptic damage. Notably, we found that PBM increased the level of GluR 1 phosphorylation and attenuated AMPAR endocytosis induced by Aβ_1‐42_, thereby alleviating synaptic dysfunction.

Taken together, the present study provides preclinical evidence that inhibition of JNK3 phosphorylation by PBM treatment can effectively rescue AMPA receptor endocytosis and dramatically reduce amyloid load, neuroinflammation, and synaptic loss in APP/PS1 transgenic mice. Moreover, our observations suggest that in both cultured primary neurons and transgenic mice, inhibiting phosphorylation of JNK3 by PBM may be an important step for the attenuation of Aβ‐induced neurotoxicity. In addition, our findings, together with previous studies, indicate that in AD experimental models, PBM activates ERK and subsequently phosphorylates and stabilize MKP7, resulting in JNK3 inactivation. Thus, additional research on the regulatory mechanism of photobiomodulation may lead to an effective therapeutic strategy to treat AD.

## 4 EXPERIMENTAL PROCEDURES

### 4.1 Transgenic mice

The transgenic mice (APP/PS1) used in this study were obtained from the Jackson Laboratory (Bar Harbor, ME, USA). See the Supporting Information for details.

### 4.2 PBM treatment

For *in vitro*, all group of cells were treated with various chemicals and/or irradiated with a semiconductor laser (635 nm, NL‐FBA‐2.0‐635, nLight Photonics Corporation, Vancouver, WA; Laser Technology Application Research Institute, Guangzhou, China) for 5 min in the dark, with corresponding radiant fluences of 2 J/cm^2^ (Table S1). The beam area at culture surface is 9.6 cm^2^. The chemicals were added to the culture medium 30 min before PBM treatment. The entire procedure was carried out at room temperature. Throughout each experiment, the cells were kept in either a complete dark or a very dim environment, except when subjected to the light irradiation, to minimize the ambient light interference. Neurons were treated with PBM again half an hour before harvesting neurons at 24 h for Western blot analysis, co‐immunocoprecipitation, PSD preparation, and again PBM treatment here was to be better detect the activation of proteins involved in PBM‐induced cell signaling pathway. For *in vivo*, after hair removal, six‐month‐old WT or APP/PS1 mouse was placed in the mouse fixator without anesthesia and only exposed their head and tail, while the optical fiber is located above the head of the mouse, and the tail does not receive light. Next, WT or APP/PS1 mouse was exposed to one of two stimulation conditions: without PBM, or with PBM (635 nm, 6 J/cm^2^) daily for 10 minutes for 30 days. The area projected on the skin surface was 0.875 cm^2^. The power to the skin surface was 92 mM, and the power density was 117.2 mW/cm^2^ (Figure S1a). The PBM system was stationary positioned in skin contact at the frontal region in the head of mice; the irradiation was applied once a day for 10 min, at the irradiation dose of 6 J/cm^2^ with power of 8.75 mW exposure to the cortex for 30 days (without local temperature increase in the scalp and brain tissue) (Luz Eltchechem et al., 2017). Refer to our previous studies on the transmittance of PBM from the upper part of the exposed brain to the interior of the hippocampus, the transmittance was approximately 30% (Zhang et al., 2020), and then, the hippocampus is required to obtain a dose of 2 J/cm^2^, which indicated that 6 J/cm^2 ^PBM irradiation is needed. A penetrating dose of 2 J/cm^2^ reaching the hippocampus was used in animal experiments, which was consistent with the dose of PBM used *in vitro*. The control groups were maintained in the same mouse fixator for the same amount of time as the irradiated groups, but the laser source was not activated. All animals were randomly divided into groups and all analyses were done blind without knowledge of the experimental manipulation that had been performed using raw images.

### 4.3 RNA interference‐mediated gene silencing

For RNAi‐mediated gene silencing, we used MKP7‐specific siRNA (designed by Genepharma) and NC siRNA. Cells were transfected with specific siRNA oligonucleotides using Lipofectamine 3000. After transfection 24 h later, cells were assayed for gene silencing by Western blot. All analyses were done blind without knowledge of the experimental manipulation that had been performed using raw images.

### 4.4 Western blot analysis and co‐immunoprecipitation

After individual incubations, cell proteins were extracted in lysis buffer (50 mM Tris‐HCl, pH 8.0, 150 mM NaCl, 1% Triton X‐100, 100 μg/ml PMSF) supplemented with protease inhibitor cocktail set I for 60 min on ice. After centrifugation (4°C, 13,201 *g*, 20 min), equivalent samples were resolved by 10% or 15% SDS‐PAGE Bis‐Tris gels and transferred to polyvinylidene difluoride (PVDF) membranes (Roche Applied Sciences, Indianapolis, IN, USA). The membranes were blocked in TBST (10 mM Tris‐HCl, pH 7.4, 150 mM NaCl, 0.1% Tween 20) containing 5% nonfat milk and then incubated with a primary antibody and a secondary antibody. The detection of signals was using an ODYSSEY Infrared Imaging System (LI‐COR) to perform. The intensity of the Western blot signals was quantified using ImageJ software (National Institutes of Health (NIH), Bethesda, MD). The densitometry analyses are presented as the ratio of protein to respective loading control protein such as GAPDH, β‐actin, or total proteins of the corresponding phosphorylated proteins, are compared with the control group, and normalized to 1.

For co‐IP, protein extracts were incubated with the indicated antibodies for 2–4 h at room temperature and then incubated with 50% slurry protein A + G Sepharose (Beyotime) at 4°C overnight. The pellet was resuspended with the same volume of SDS sample buffer and boiled 7 min to remove protein A + G Sepharose beads. Then, the whole cells lysates and immunoprecipitates were all analyzed by Western blot analysis. For detecting PSD95 phosphorylation, in brief, the lysates from primary neurons were subjected to immunoprecipitation with PSD95 antibody, and the immune complexes were separated with 10% gradient SDS‐PAGE gel and analyzed by immunoblotting using anti‐phosphoserine antibody and PSD95 antibody. All analyses were done blind without knowledge of the experimental manipulation that had been performed using raw images.

### 4.5 Enzyme‐linked immunosorbent assay for Aβ

The cerebral cortex was isolated from mice and subjected to Aβ measurement with the use of Aβ_1‐40_ or Aβ_1‐42_ enzyme‐linked immunosorbent assay kit according to the manufacturer's instructions (Invitrogen, USA). The soluble Aβ fraction probably contained monomeric and oligomeric Aβ. Insoluble Aβ was treated with 5 M guanidine/50 mM Tris‐HCL (pH 8.0) buffer before ELISA measurement (Martorell et al., 2019).

### 4.6 Immunocytochemistry

To assay total AMPA receptor endocytosis, we followed the method described previously (Bhattacharyya et al., 2009), and surface AMPARs were labeled in live neurons by 15‐min incubation at 37°C with a rabbit monoclonal AMPAR antibody directed against the N terminus of the GluR 1 subunit (1:200 in conditioned media). After washout of the antibodies, cells were given appropriate drug and PBM treatments and then were further incubated for 30 min. Following washing out the drug, cells were chilled on ice, and antibodies remaining on surface receptors were stripped with an acidic solution (0.5 M NaCl, 0.2 N acetic acid) for 3 min on ice. Cells were then fixed in 4% paraformaldehyde (PFA), permeabilized with 0.2% Triton X‐100 for 30 min at room temperature, and stained with goat anti rabbit Alexa‐555 secondary antibody (Abcam). Phalloidin staining was used to label the morphology of neurons. After five additional washes with PBS, internal AMPAR was analyzed by confocal microscopy (LSM 510 META; Carl Zeiss MicroImaging) and LSM 510 META software (Carl Zeiss MicroImaging). All analyses were done blind without knowledge of the experimental manipulation that had been performed using raw images. Images from each experiment were thresholded using identical values for different experimental conditions, and the total thresholded area of fluorescently labeled internalized GluR1 intensity was measured using ImageJ (NIH). These values were then normalized to the average internalized fluorescence of untreated control group.

### 4.7 Immunohistochemistry

For histological processing, we followed the method described previously (Zhao et al., 2017). After behavioral testing, all mice were deeply anesthetized with sodium pentobarbital (50 mg/kg, i.p), and mice were perfused transcardially with ice‐cold PBS. The right hemispheres of brains were lysed for protein extraction, and the left hemisphere was dissected and post‐fixed in 4% PFA in 0.1 M PBS, then dehydrated in 15% and 30% sucrose solution at 4^o^C for histological processing. The brains were embedded in Optimal Cutting Temperature (OCT) compound, and sequential coronal brain sections (10‐μm thick) were obtained using a freezing microtome (Leica, CM1850) and mounted on polylysine‐coated slides (Sigma‐Aldrich). Sections were incubated at 80°C for 10 min in 10 mM tri‐sodium citrate, pH 8.5, in water, and then after cooled to room temperature, permeabilized, and blocked in PBS with 0.2% Triton X‐100 for 45 min and 3% BSA at room temperature for 1 h. The sections were incubated in appropriately diluted primary antibodies containing PBS with 0.2% Triton X‐100 at 4°C overnight. Primary antibodies were detected with Alexa Fluor 488/555‐conjugated secondary antibodies. Anti‐Aβ antibody for immunohistochemistry was purchased from Biolegend. Immunofluorescence images of cerebral cortex and hippocampus in different treatment groups were obtained by laser scanning confocal microscopy (Zeiss LSM 510 META or Zeiss LSM 880). In image acquisition, we follow the principle that different treatment groups select the same hippocampal region, as well as the similar cerebral cortex region, to avoid regional differences. The mean fluorescence intensity of each protein was quantified by ImageJ (NIH) software with a set threshold and normalized to the control group. Images from each experiment were thresholded using identical values for different experimental conditions. The "analyze particles" function in ImageJ was used for counting amyloid load area, and a set threshold was used for both control and experimental groups. For histology analysis, after incubating with primary antibodies, sections then incubated with goat anti‐mouse immunoglobulin G (IgG) horseradish peroxidase (HRP) (1:50, Beyotime). Diaminobenzidine (DAB; Beyotime) was used as the chromagen and hematoxylin (C0105; Beyotime) was used as the counterstain (Zhao et al., 2017). Sections were stained with thioflavin T, a highly sensitive marker of Aβ deposits, for 8 min at room temperature. All analyses were done blind without knowledge of the experimental manipulation that had been performed using raw images.

### 4.8 Phalloidin staining

It has been reported that phalloidin can be used to mark dendrites and dendritic spines (Tada et al., 2007). FITC phalloidin is an actin green fluorescent probe. Cells were incubated at room temperature with FITC phalloidin (1:50 diluted in PBS) for 45 min. FITC‐labeled phalloidin was viewed under a confocal microscope. All analyses were done blind without knowledge of the experimental manipulation that had been performed using raw images.

### 4.9 Behavior tests

In Y‐maze test, all apparatus and objects were cleaned with 70% ethanol before and after each trial. The maze was placed in a separate room with a light. The floor of the maze was covered with sawdust, which was mixed after each individual trial in order to eliminate olfactory stimuli. The mice were placed in the end of one arm (30 cm length, 10 cm height) and allowed to move freely for 7 min. The percentage spontaneous alternation was calculated as the ratio of the number of successful alternations to possible alternations (defined as the total number of arm entries minus two), multiplied by 100 (Yan et al., 2001).

The Y‐maze test consisted of two trials separated by an intertrial interval (ITI) to assess spatial recognition memory (Ma et al., 2007). The first trial (training) had 5‐min duration and allowed the mouse to explore only two arms (start arm and other arm) of the maze, with the third arm (novel arm) being blocked. After 2 h, the second trial (recall period) was conducted. For the second trial, the mouse was placed back in the maze in the same starting arm, with free access to all three arms for 3 min. All trials were recorded by CCD camera. Video recordings were later analyzed, and the distance in novel arm, the number of entries, time spent in each arm, and the average speed were analyzed. All analyses were done blind without knowledge of the experimental manipulation that had been performed using raw data.

Morris Water Maze (MWM) Test was performed as previously described (Martorell et al., 2019; Zhang et al., 2020) to evaluate spatial learning and memory abilities after PBM treatment. Spatial reference memory testing was performed in a circular tank (diameter, 1.2 m) filled with white opaque water at approximately 22°C. Reference tips consisting of different colors and shapes are placed along the walls around the tank. Inside the tank is a fixed platform (diameter, 10 cm) located in the target quadrant. Mice were provided 60 s to search for the platform, which if not found, were gently guided to it. Animals were kept on the platform for 15 s. Four trials were conducted daily with 1 h intertrial interval. During the training trials, mice were released into the maze from a randomly selected quadrant, with all animals using the same order. Between trials, the mice were gently wiped dry and placed in a warmer place. Escape latency to find the platform was recorded and averaged per testing day of each mouse. On day 6, the platform was removed and a probe trial (memory test) was performed. The time spent in each of the 4 quadrants and the number of crossing of the area where the platform used to be was recorded by a video camera linked to a computer‐based image analyzer. Swimming speed was recorded automatically.

### 4.10 Statistical analysis

Data are from one representative experiment among at least three independent experiments and are expressed as the mean ± SEM. Statistical analysis was performed using SPSS software by Student's *t* test for experiments with two groups, and experiments with more than two groups was subjected to one‐way ANOVA or two‐way ANOVA followed by Tukey's *post hoc* tests for multiple comparisons. And the differences were considered statistically significant at *p < *0.05. Specific statistical parameters are detailed in the figure legends.

## CONFLICT OF INTEREST

The authors declare that they have no conflict of interest.

## AUTHOR CONTRIBUTIONS

Q.S., L.L., and D.X. conceived of and designed the research. Q.S. and X‐T. G., performed experiments and analyzed data. Q.S., L.L., and D.X. interpreted experimental results and drafted the manuscript. Q.S., L.L., X‐T. G., and D.X. revised and approved the final version of manuscript.

## ETHICAL APPROVAL

The present study was performed in accordance with the guidelines of the Guide for the Care and Use of Laboratory Animals (Institute of Laboratory Animal Resources, Commission on Life Sciences, National Research Council). It was approved by the Institutional Animal Care and Use Committee of our university (South China Normal University, Guangzhou, China).

## Supporting information

Figure S1‐S10‐Table S1

## Data Availability

All data generated or analyzed during this study are included in this submitted article and its additional files.
