# Correction to “Photobiomodulation Suppresses JNK3 by Activation of ERK/MKP7 to Attenuate AMPA Receptor Endocytosis in Alzheimer's Disease”

**DOI:** 10.1111/acel.70404

**Published:** 2026-02-06

Shen, Q., Liu, L., Gu, X. and Xing, D. (2021) Photobiomodulation Suppresses JNK3 by Activation of ERK/MKP7 to Attenuate AMPA Receptor Endocytosis in Alzheimer's Disease. *Aging Cell*, 20: e13289. https://doi.org/10.1111/acel.13289.

In the originally published version of this article, an error occurred in the assembly of Figure 3h. Specifically, an incorrect representative immunohistochemistry image was inadvertently included for the APP/PS1+PBM group. This correction is limited to the representative image presentation and does not affect the conclusions of the study. As a necessary effort, the authors have now corrected this figure.

The correct figure is shown below.

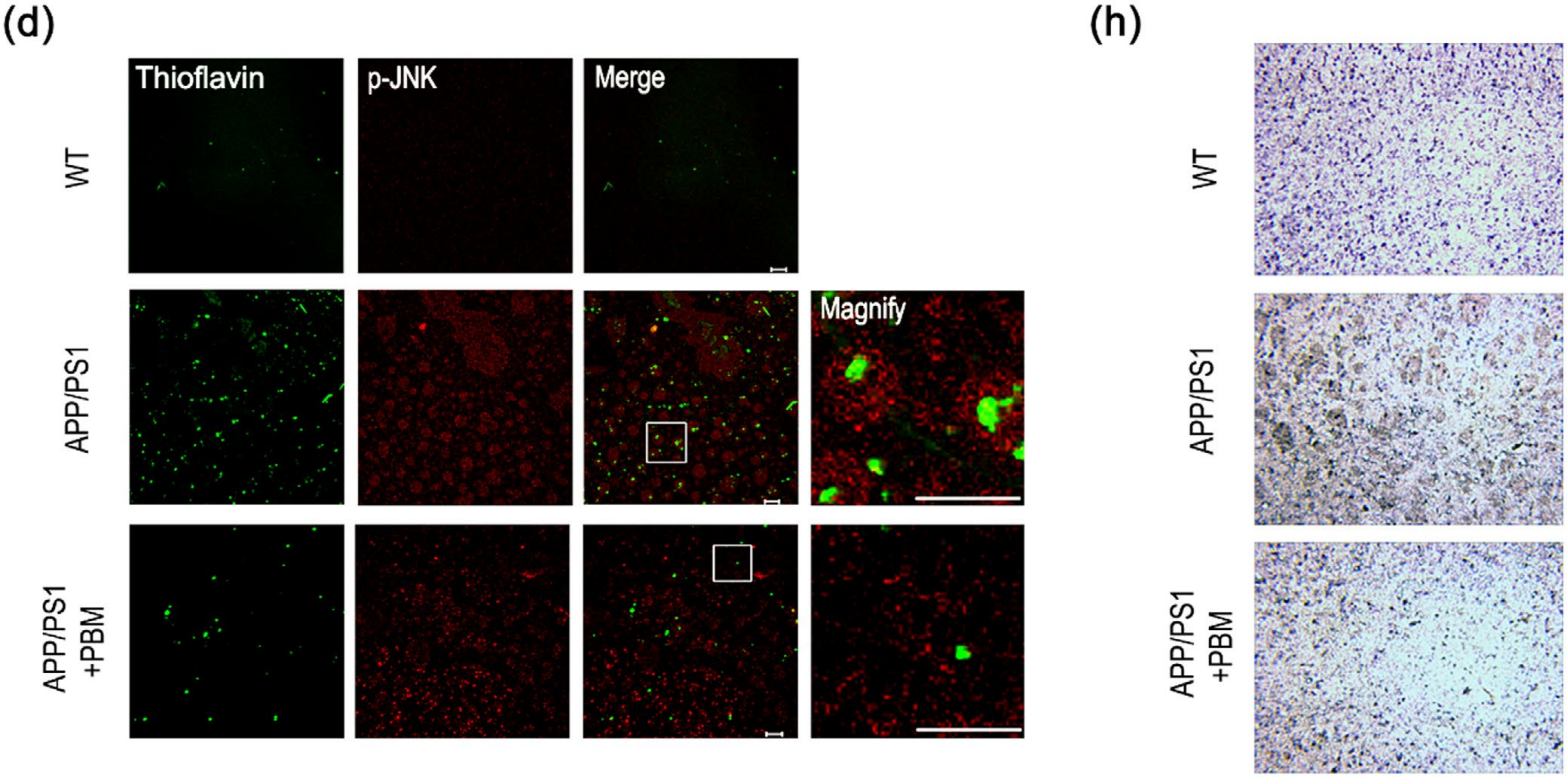

We apologize for this error.

