# Supplementary material for: Photobiomodulation suppresses JNK3 by activation of ERK/MKP7 to attenuate AMPA receptor endocytosis in Alzheimer's disease
Source: Aging Cell. 2020 Dec 18;20(1):e13289. doi: 10.1111/acel.13289 (PMC7811840; doi:10.1111/acel.13289)
Supplement: Supplementary file 1 — Figure S1‐S10‐Table S1 [file ACEL-20-e13289-s001.doc]

Supporting information for

**Photobiomodulation Suppresses JNK3 by Activation of ERK/MKP7 to Attenuate AMPA Receptor Endocytosis in Alzheimer's Disease**

Qi Shen^1,2^, Lei Liu^2^, Xiaotong Gu^1,2^, Da Xing^1,2^*

^1^MOE Key Laboratory of Laser Life Science & Institute of Laser Life Science, South China Normal University, Guangzhou 510631, China

^2^College of Biophotonics, South China Normal University, Guangzhou 510631, China

*To whom correspondence and proof should be addressed

Da Xing, Ph.D. E-mail: xingda@scnu.edu.cn

**Experimental procedures**

**Materials**

| **REAGENT OR RESOURCE** | **SOURCE** | **IDENTIFIER** |
| --- | --- | --- |
| Antibodies | | |
| Anti-Glutamate Receptor 1 (AMPA subtype) (phospho S845) antibody | Abcam | Cat# ab3901 |
| AMPA Receptor (GluR 1) (D4N9V) | Cell Signaling Technology | Cat# 13185 |
| JNK3 (55A8) | Cell Signaling Technology | Cat# 2305 |
| DUSP16/MKP7 (D5F4) | Cell Signaling Technology | Cat# 5523 |
| Phospho-SAPK/JNK | Cell Signaling Technology | Cat# 9255 |
| Phospho-c-Jun (Ser 63) | Cell Signaling Technology | Cat# 9261 |
| Phospho-p44/42 MAPK (Erk1/2) (Thr202/Tyr204) | Cell Signaling Technology | Cat# 4370 |
| p44/42 MAPK (Erk1/2) | Cell Signaling Technology | Cat# 4695 |
| Phospho-APP (Thr668) | Cell Signaling Technology | Cat# 6986 |
| Anti-Phosphoserine | Merck Millipore | Cat# 05-1000 |
| PSD95 | Proteintech | Cat# 20665-1-AP |
| MAP2 | Proteintech | Cat# 17490-1-AP |
| Synaptophysin | Proteintech | Cat# 60191-1-lg |
| GFAP | Cell Signaling Technology | Cat# 3670 |
| Iba1 | Proteintech | Cat# 10904-1-AP |
| GAPDH | Santa Cruz Biotechnology | Cat# sc-32233 |
| β-actin | Santa Cruz Biotechnology | Cat# sc-47778 |
| β-amyloid | Santa Cruz Biotechnology | Cat# sc-28365 |
| Aβ (6E10) (for immunohistochemistry) | Biolegend | Cat# 803015 |
| Goat Anti-Mouse IgG H&L (Alexa Fluor 488) | Abcam | Cat# ab150113 |
| Goat Anti-Mouse IgG H&L (Alexa Fluor 555) | Abcam | Cat# ab150114 |
| Goat Anti-Rabbit IgG H&L (Alexa Fluor 488) | Abcam | Cat# ab150077 |
| Goat Anti-Mouse IgG H&L (Alexa Fluor 680) | Abcam | Cat# ab175775 |
| Goat Anti-Rabbit IgG H&L (Alexa Fluor 790) | Abcam | Cat# ab175781 |
| Goat Anti-Mouse IgG H&L (Alexa Fluor 790) | Abcam | Cat# ab175783 |
| Goat Anti-Rabbit IgG H&L (Alexa Fluor 680) | Abcam | Cat# ab175773 |
| HRP-labeled Goat Anti-Mouse IgG(H+L) | Beyotime | Cat# A0216 |
| Chemicals, Peptides | | |
| Small interfering RNA | GenePharma | N/A |
| β-Amyloid_1-42_ (human) | Invitrogen | Cat# 03-112 |
| Lipofectamine 3000 | Invitrogen | Cat# L3000015 |
| PD98059 | Gibco | Cat# PHZ1164 |
| API-2 | Santa Cruz Biotechnology | Cat# sc-200661 |
| FITC-phalloidin | Beyotime | Cat# C1033 |
| Dulbecco's Modified Eagle's medium | Gibco | Cat# 12800017 |
| Neurobasal Medium | Gibco | Cat# 12349-015 |
| B27 | Gibco | Cat# 17504-044 |
| L-Glutamine | Gibco | Cat# A2916801 |
| poly-L lysine | Sigma | Cat# p6282 |
| 0.25% Trypsin-EDTA | Gibco | Cat# 25200072 |
| HBSS | Gibco | Cat# 14185052 |
| Fetal Bovine Serum | Gibco | Cat# 10099133C |
| polyvinylidene difluoride (PVDF) membranes | Roche | Cat# 03010040001 |
| SP600125 | MedChemExpress | Cat# HY-12041 |
| Dimethylsulfoxide | Sigma | Cat# D2650-100 ml |
| Thioflavin T | Sigma | Cat# T3516 |
| Diaminobenzidine (DAB) Horseradish Peroxidase Color Development Kit | Beyotime | Cat# P0203 |
| Hematoxylin | Beyotime | Cat# C0105 |
| Protein A+G Agarose | Beyotime | Cat# P2012 |
| FD Rapid GolgiStain^TM^ Kit | FD Neurotechnologies | Cat# PK401 |
| Human Aβ_1-40_ and Aβ_1-42_ ELISA kits | Invitrogen | Cat# KHB3481  Cat# KHB3442 |

**Animals**

The characterization of the APP/PS1 double transgenic mice, expressing a chimeric mouse/human amyloid precursor protein bearing the Swedish mutation (Mo/HuAPP695swe) and a mutant human Presenilin 1 protein (PS1-dE9) in central nervous system neurons . In this study, 3-month-old, 6-month-old APP/PS1 transgenic mice and age-matched WT mice were used (12-14 mice in each group). For SP600125 treatment, 6-month-old APP/PS1 mice were randomized into 4 groups (*n* = 7, each group): vehicle-treated APP/PS1 mice, vehicle-treated APP/PS1 mice with PBM, SP600125-treated APP/PS1 mice, SP600125-treated APP/PS1 mice with PBM. Each mouse with SP600125 treatment received SP600125 dissolved in 2% dimethylsulfoxide (DMSO) in PBS at a dose of 30 mg/kg body weight once per day by intraperitoneal injection (i.p) for 30 days, whereas each mouse with vehicle treatment received an equal volume of 2% DMSO in PBS as control. The dose of SP600125 in this study was selected based on previously published studies . The mice were housed in individual cages in a controlled environment (constant temperature 22 + 1^o^C, humidity 50-60%, lights on 07:00-19:00 h). Animals had food and water available *ad* *libitum*. In experiments, the same batch of mice was randomly divided into different groups.

**Golgi-Cox staining**

Brains were stained using FD Rapid GolgiStain^TM^ Kit (FD Neurotechnologies, PK401) according to the manufacturer's instruction. Briefly, the brain tissues were rinsed with Milli Q water to remove blood from the surface. Mouse brain tissues were impregnated in equal volumes of solutions A and B (equal volumes of solutions A and B need to be prepared one day in advance), and the impregnation solution was replaced the next day and stored in the dark at room temperature for 2 weeks. After 2 weeks, the brain tissues were transferred to solution C for 72 h in the dark. The brain sections (100 μm thickness) were generated using a freezing microtome (Leica, CM1850) with the chamber temperature set at -22^o^C. Each slice was mounted on a gelatin-coated slide using solution C as previously described , and allowed to dry naturally at room temperature for at least 1 day. Next, the dried brain sections were stained as described in the product manual. The dendrites were imaged using 40× objectives using inverted microscope (MSHOT, MF53) and the counting was performed using Image J (Analysis Skeleton Plugins) software.

**Oligomer Aβ_1-42_ preparation**

Dissolved peptides were sonicated for 30 seconds and diluted in phosphate buffered saline (PBS; 1.06 mM KH2PO4, 155.17 mM NaCl, 2.97 mM Na2HPO4-7H2O, pH7.4) to the final concentration of 100 µM to make freshly prepared Aβ peptides (Mono-Aβ_1-42_). The peptides were then incubated at 22^o^C for 16 h followed by 24 h incubation at 4^o^C, centrifuged at 16,000 × g for 15 min, and the supernatant was collected as oligomerized Aβ peptides (Oligo-Aβ_1-42_) . The concentration of Aβ_1-42_ was 1 μM.

**Cell culture**

SH-SY5Y cells were cultured in Dulbecco's Modified Eagle's medium supplemented with 10% fetal bovine serum, 1 U/mL penicillin, and 1 μg/mL streptomycin in a humidified (5% CO_2_, 37^o^C) incubator.

Hippocampus was dissected from C57BL/6 mice embryonic day 14 (E14), trypsinized the tissue at 37^o^C for 30 min, and then gently dissociate into single cell suspension in culture medium containing 10% heat-inactivated fetal bovine serum, before centrifuging at 1500 rpm for 5 min. Cells were resuspended in Neurobasal Medium supplemented with B27 and L-glutamine, and seeded to a density 6 × 10^5^ viable cells/35 mm culture dishes previously coated with poly-L lysine (0.1 g/ml) for at least 3 h at 37^o^C . APP/PS1 transgenic mice neuronal cultures were used for 14 d *in vitro* (DIV).

**Cell viability assay and cell apoptosis assays**

Cell viability was assessed with CCK-8 (Dojindo Laboratories) after Aβ_1-42_ and/or PBM. At the indicated time, CCK-8 was added and incubated for 1.5 h. OD 450, the absorbance value at 450 nm, was read with a 96-well plate reader (DG5032; Huadong).

Quantification of apoptosis by Annexin-V/PI staining was performed as described previously. Apoptosis cell death was determined using the BD ApoAlert Annexin-V-FITC Apoptosis Kit (Becton Dickinson, Biosciences) according to the manufacturer's instructions. Flow cytometry was performed on a BD FACSCanto II flow cytometer (Becton Dickinson).

All analyses were done blind without knowledge of the experimental manipulation that had been performed using raw data.

**Analysis of spine density**

Dendritic spines of hippocampal neurons under different treatments were identified and counted off-line in maximum-intensity projections of the *z*-stacks using Image J software . Care was taken to ensure that each spine was counted only once by following its projection course through the stack of *z*-sections. Spines were counted only if they appeared continuous with the parent dendrite. Spine density was calculated by quantifying the number of spines per dendritic segment, and normalized to 1-μm dendrite length. All analyses were done blind without knowledge of the experimental manipulation that had been performed using raw images.

**PSD preparation**

For sub-synaptic fractionation of PSD , cultured cortical neurons at a density of at least 10 × 10^6^ cells or dorsal hippocampus tissue was homogenized with a Dounce tissue grinder in homogenization buffer containing (in mM) 320 sucrose, 10 Tris-HCl pH 7.4, 1 EDTA, 1 NaHCO_3_, 1 PMSF, 1 sodium orthovanadate, 5 NaF, 20 β-glycerophosphate, and protease inhibitor cocktail. The homogenate was centrifuged at 1,000 × *g* (10 min), and the supernatant was again centrifuged at 10,000 × *g* (15 min). The pellet was homogenized in homogenization buffer containing 0.5% Triton X-100 with a Dounce tissue grinder, incubated 40 min on ice and centrifuged at 32,000 × *g* (20 min). The resulting pellet containing PSDs was processed for protein extraction. Briefly, the pellet was resuspended in RIPA buffer on ice for 20 min. The samples were centrifuged at 11,500 × *g* for 10 min and the protein concentration of the supernatant was determined by the Bradford method.

**References**

Boros BD, Greathouse KM, Gentry EG, Curtis KA, Birchall EL, Gearing M, Herskowitz JH (2017). Dendritic spines provide cognitive resilience against Alzheimer's disease. *Annals of Neurology*. **82**, 602-614.

Das G, Reuhl K, Zhou R (2013). The Golgi-Cox method. *Methods in Molecular Biology*. **1018**, 313-321.

Jankowsky JL, Fadale DJ, Anderson J, Xu GM, Gonzales V, Jenkins NA, Copeland NG, Lee MK, Younkin LH, Wagner SL, Younkin SG, Borchelt DR (2003). Mutant presenilins specifically elevate the levels of the 42 residue β-amyloid peptide in vivo: evidence for augmentation of a 42-specific γ secretase. *Human Molecular Genetics*. **13**, 159-170.

Kim T, Vidal GS, Djurisic M, William CM, Birnbaum ME, Garcia KC, Hyman BT, Shatz CJ (2013). Human LilrB2 Is a β-Amyloid Receptor and Its Murine Homolog PirB Regulates Synaptic Plasticity in an Alzheimer’s Model. *Science*. **341**, 1399.

Manczak M, Kandimalla R, Yin X, Reddy PH (2018). Hippocampal mutant APP and amyloid beta-induced cognitive decline, dendritic spine loss, defective autophagy, mitophagy and mitochondrial abnormalities in a mouse model of Alzheimer's disease. *Human Molecular Genetics*. **27**, 1332-1342.

Nobili A, Latagliata EC, Viscomi MT, Cavallucci V, Cutuli D, Giacovazzo G, Krashia P, Rizzo FR, Marino R, Federici M, De Bartolo P, Aversa D, Dell’Acqua MC, Cordella A, Sancandi M, Keller F, Petrosini L, Puglisi-Allegra S, Mercuri NB, Coccurello R, Berretta N, D’Amelio M (2017). Dopamine neuronal loss contributes to memory and reward dysfunction in a model of Alzheimer’s disease. *Nature Communications*. **8**, 14727.

Poppe L, Rué L, Timmers M, Lenaerts A, Storm A, Callaerts-Vegh Z, Courtand G, de Boer A, Smolders S, Van Damme P, Van Den Bosch L, D’Hooge R, De Strooper B, Robberecht W, Lemmens R (2019). EphA4 loss improves social memory performance and alters dendritic spine morphology without changes in amyloid pathology in a mouse model of Alzheimer’s disease. *Alzheimer's Research & Therapy*. **11**, 102.

Schnell E, Long TH, Bensen AL, Washburn EK, Westbrook GL (2014). Neuroligin-1 knockdown reduces survival of adult-generated newborn hippocampal neurons. *Frontiers in Neuroscience*. **8**, 71.

Yang L, Wang Z, Wang B, Justice NJ, Zheng H (2009). Amyloid precursor protein regulates Cav1.2 L-type calcium channel levels and function to influence GABAergic short-term plasticity. *The Journal of Neuroscience* **29**, 15660-15668.

Zhou Q, Wang M, Du Y, Zhang W, Bai M, Zhang Z, Li Z, Miao J (2015). Inhibition of c-Jun N-terminal kinase activation reverses Alzheimer disease phenotypes in APPswe/PS1dE9 mice. *Annals of Neurology*. **77**, 637-654.

| **Table S1** | | | |
| --- | --- | --- | --- |
| **A** |  |  |  |
|  | Irradiation Parameters | |  |
|  | Parameter [unit] | Semiconductor laser |  |
|  | Center wavelengh [nm] | 635 |  |
|  | Operating mode | Continuous wave |  |
|  | Peak radiant power [W] | 2 |  |
|  | Aperture diameter [cm] | 1 |  |
|  | Irradiance at aperture [W/cm^2^] | 2.55 |  |
|  |  |  |  |
| **B** |  |  |  |
|  | Treatment Parameters | | |
|  | Parameter [unit] | *in vivo* | *in vitro* |
|  | Beam area at target [cm^2^] | 0.875 | 9.6 |
|  | Irradiance at target [mW/cm^2^] | 10 | 6.67 |
|  | Exposure duration [sec] | 600 | 300 |
|  | Radiant exposure [J/cm^2^] | 6 | 2 |
|  | Number and frequency of treatment sessions | Once a day, last for a month | One time in each experiment |

**Table S1.** Irradiation and treatment parameters for photobiomodulation (PBM) therapy.

**
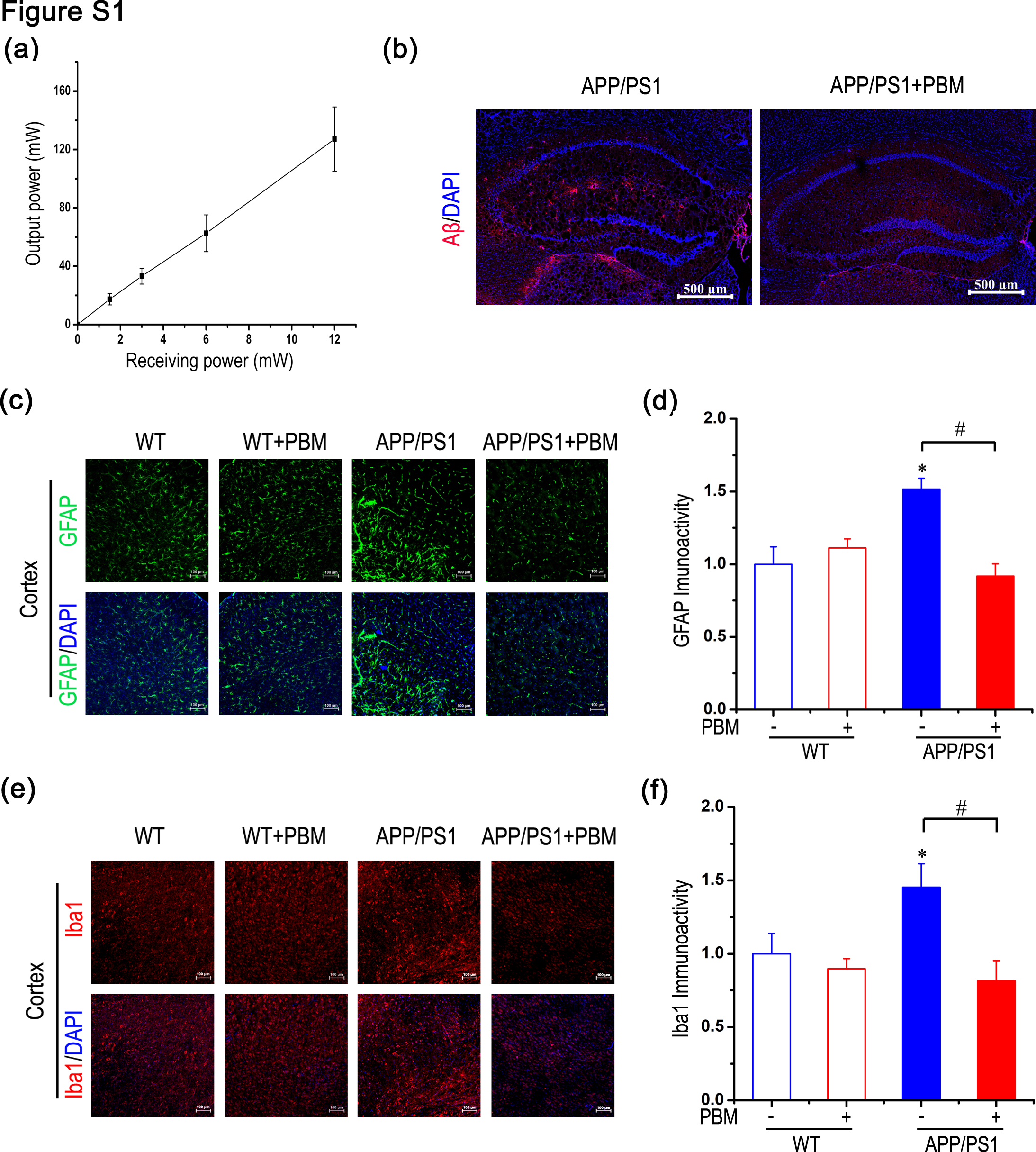
**

**Figure S1.** Effects of PBM on Aβ load and neuroinflammation in APP/PS1 mice. (a) Analysis of laser output power and cerebral cortex receiving power. The transgenic mice (APP/PS1) used in this study were produced by coinjection with the APPswe and PS1dE9 vectors. The transgenic mice were irradiated with a semiconductor laser (635 nm, NL-FBA-2.0-635, nLight Photonics Corporation, Vancouver, WA; Laser Technology Application Research Institute, Guangzhou, China) for 10 min in the dark, with the corresponding fluences of 6 J/cm^2^. We measured the laser output power and

cortex receiving power by using a power meter, and the penetration rate is 9.5%. (b) Immunohistochemistry with anti-Aβ (6E10) in the whole hippocampal region of APP/PS1 mice with or without PBM, Nuclei were counterstained with DAPI (blue), scale bar: 500 μm. *n* = 4-5 mice/group, at least 3 individual experiments. (c) Representative immunofluorescent images of GFAP (green) staining taken from cortex region of each group. Nuclei were counterstained with DAPI (blue). Scale bar, 100 μm. (d) The immunoactivity associated with GFAP in each group was further quantified and normalized to WT group (*n* = 4-5 for each group, at least 3 individual experiments, mean + SEM, Two-way ANOVA, **p* < 0.05 vs. WT group; #*p* < 0.05 vs. indicated group). (e) Representative immunofluorescent images of Iba1 (red) in the cortex region of each group, nuclei were counterstained with DAPI (blue). Scale bar, 100 μm. (f) Quantification of (e). The results are presented as means + SEM for 4-5 animals each group. **p* < 0.05 vs. control group; #*p* < 0.05 vs. indicated group.


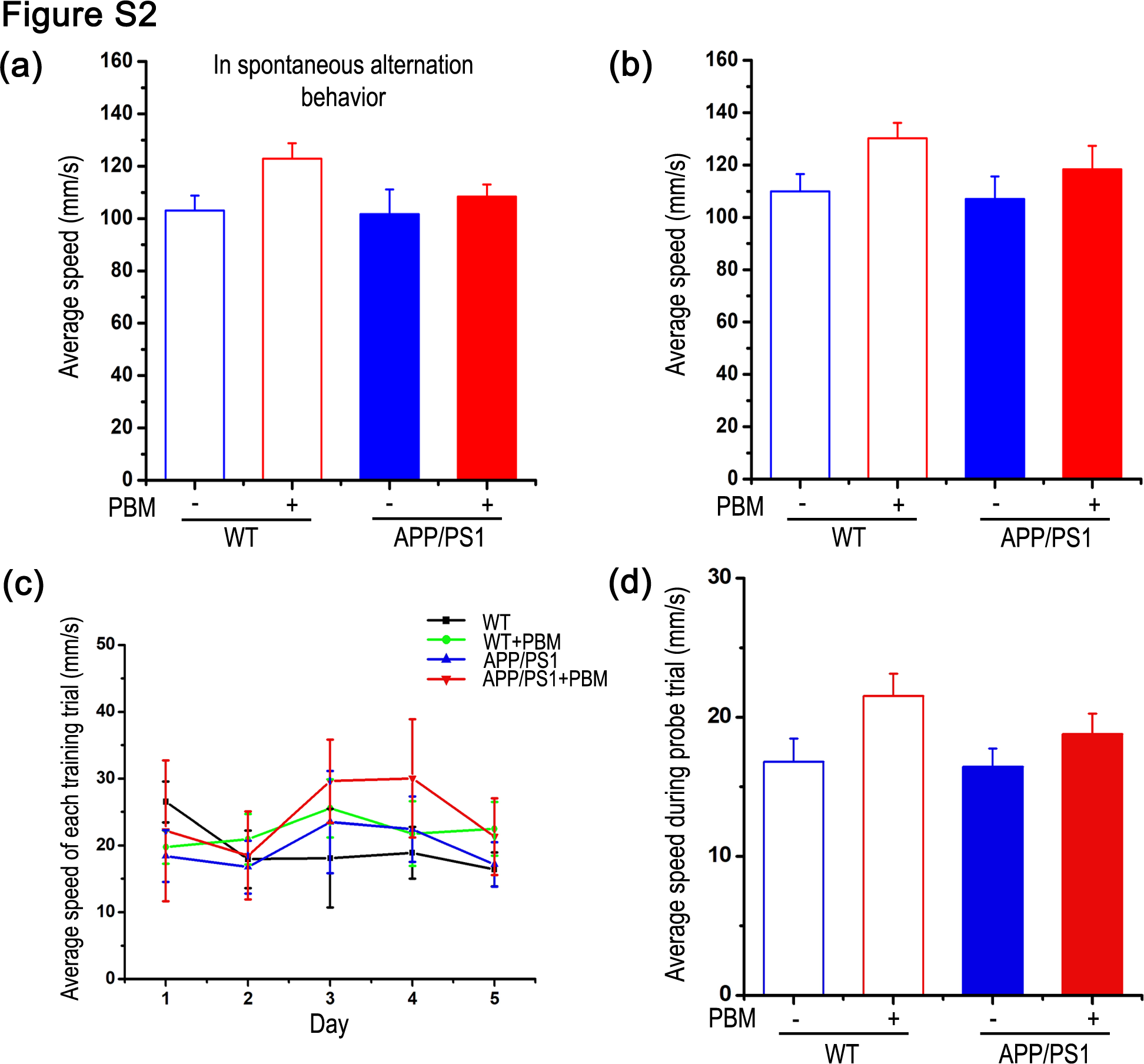


**Figure S2.** Effects of PBM on the average speed in the Y-maze task and Morris water maze task. (a) For spontaneous alternation behavior, the average speed of each group was measured. (b) The average speed of each group during the recall period (second trail) of Y-maze task was recorded. (c) The average swimming speed of each training trial day. (d) The average swimming speed during probe trial. All data are presented as mean + SEM from 12-14 mice in each group.

**
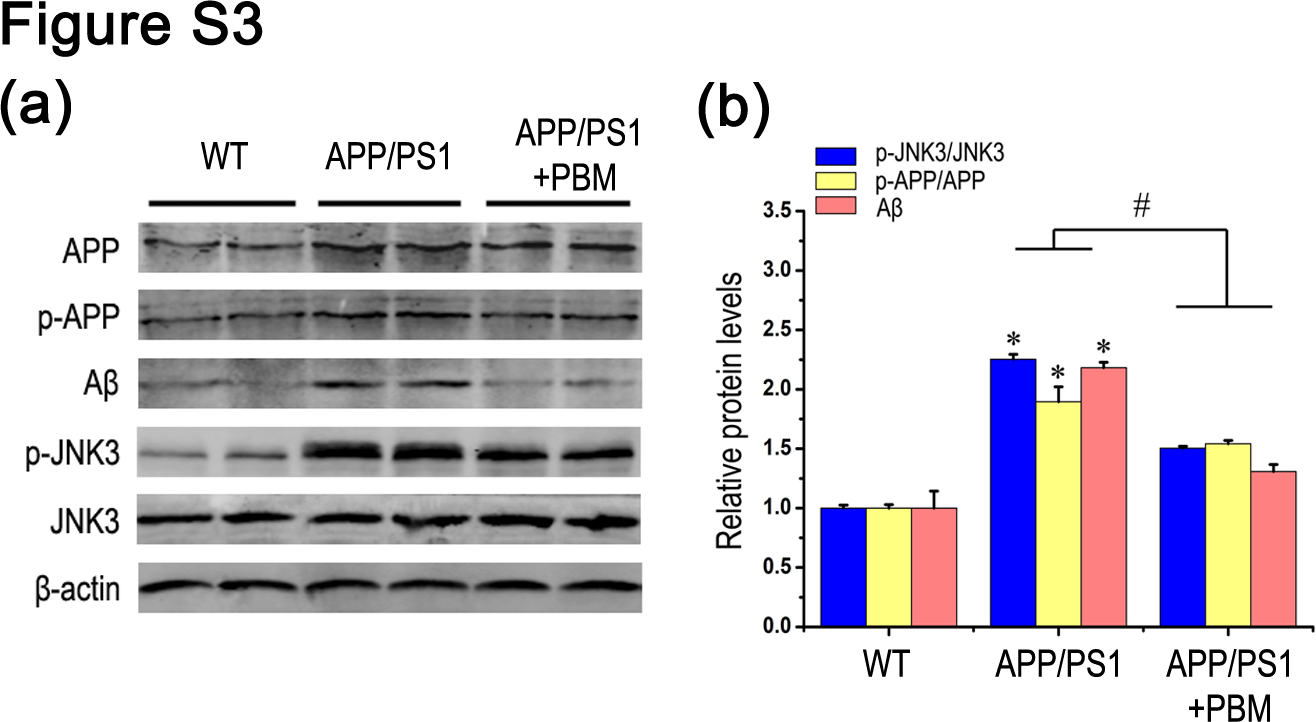
**

**Figure S3.** PBM inhibits JNK3 phosphorylation and APP Thr668 phosphorylation in an AD transgenic mouse model. (a-b) Western blot analysis of cortex lysates from APP/PS1 transgenic mice with or without PBM (6 J/cm^2^) and age matched WT mice. All the data are presented as means + SEM (*n* = 4-5 mice for each group), at least 3 individual experiments. **p <* 0.05 vs. control group; #*p <* 0.05 vs. indicated group.

**
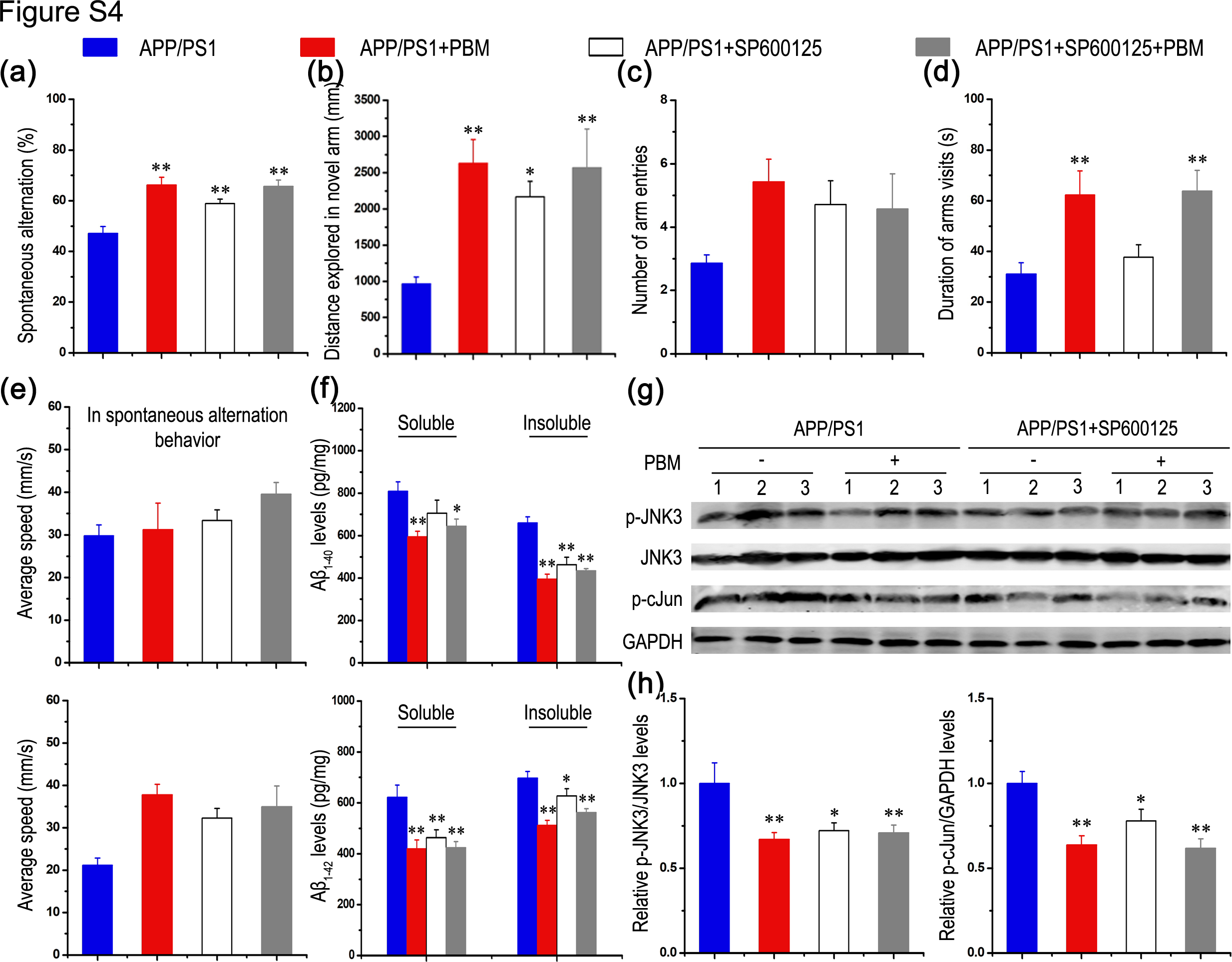
**

Figure S4. The potential disease-modifying therapeutic mechanism of PBM on memory impairment and Aβ load, which is likely achieved by regulate JNK3, in APP/PS1 mice. Y-maze task: (a) Spontaneous alternation behavior was measured in APP/PS1 group treated with PBM and/or SP600125 (30 mg/kg, i.p). (b-d) The distance explored (b), duration time (d) in novel arm and number of arm entries (c) of each group were measured. (e) The average speed of each group was measured. All data are presented as mean + SEM from 7 mice in each group. *p < 0.05 vs. APP/PS1 group, **p < 0.01 vs. APP/PS1 group by Two-way ANOVA. (f) Soluble and insoluble Aβ_1-40_ or Aβ_1-42_ levels in different groups. The Aβ measurements were performed by ELISA (n = 6 for each group, mean + SEM, Two-way ANOVA, *p < 0.05 vs. control transgenic group; **p < 0.01 vs. control transgenic group). (g-h) Representative western blot assay of p-JNK3 and p-cJun in cerebral cortex from APP/PS1 mice treated with PBM and/or SP600125 (n = 6 for each group, mean + SEM, Two-way ANOVA, *p < 0.05 vs. control transgenic group; **p < 0.01 vs. control transgenic group).

**
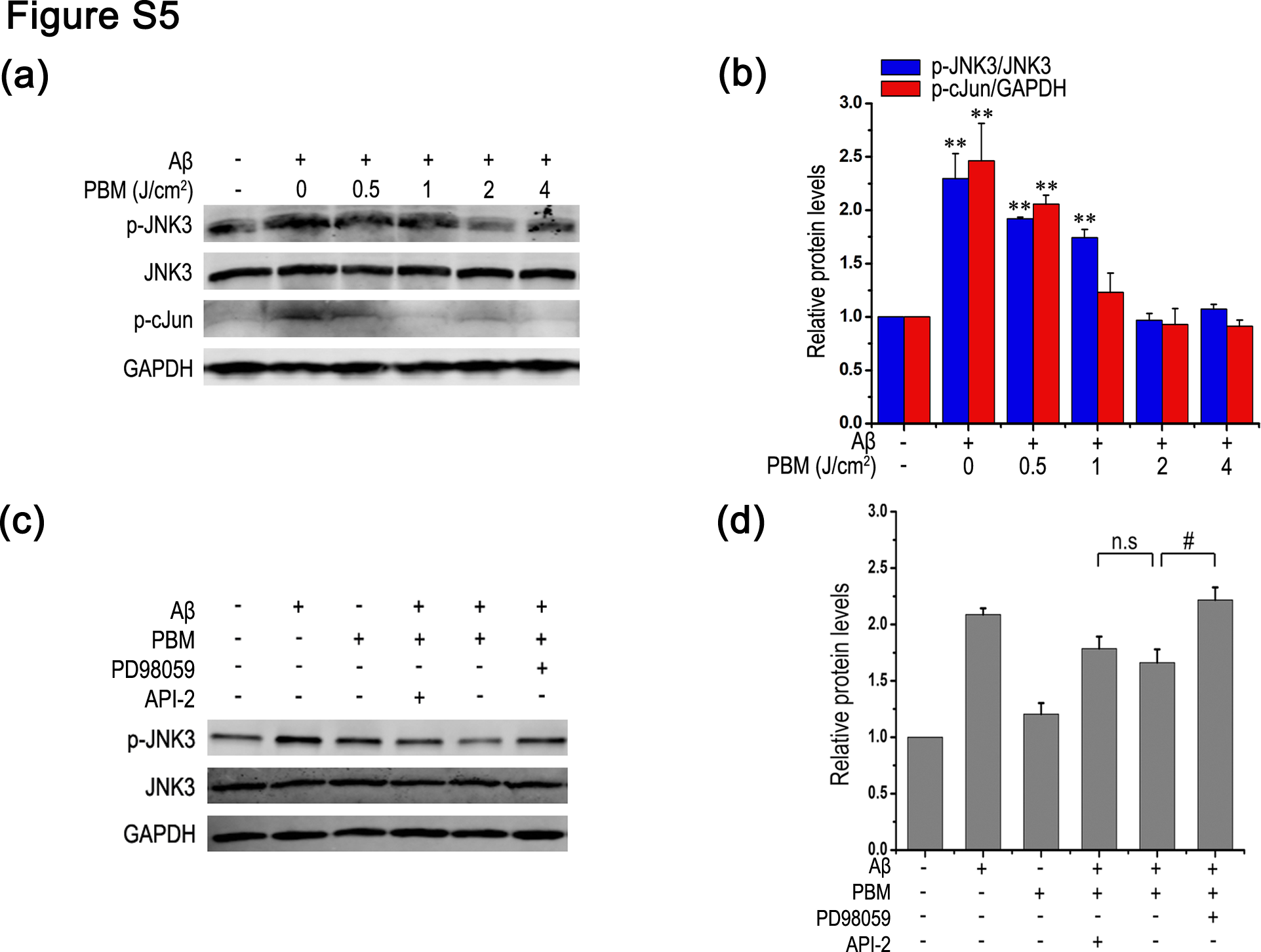
**

**Figure S5.** PBM inhibits phosphorylated form of JNK3 in Aβ-treated primary neurons through ERK-mediated signaling pathway. (a-b) Representative western blot assay for detecting dose response of PBM (0.5, 1, 2, 4 J/cm^2^) on p-JNK3, JNK3, and p-c-Jun levels in primary neurons exposed to Aβ_1-42_. (c-d) Representative western blot assay of JNK3 phosphorylation stimulated with Aβ_1-42_ and/or PBM in the presence of PD98059 (1 μM) or API-2 (2 μM) in primary neurons. All the data are presented as means + SEM for three individual experiments. **p* < 0.05 vs. control group; ***p* < 0.01 vs. control group; #*p <* 0.05 vs. indicated group; n.s indicates *p* > 0.05.


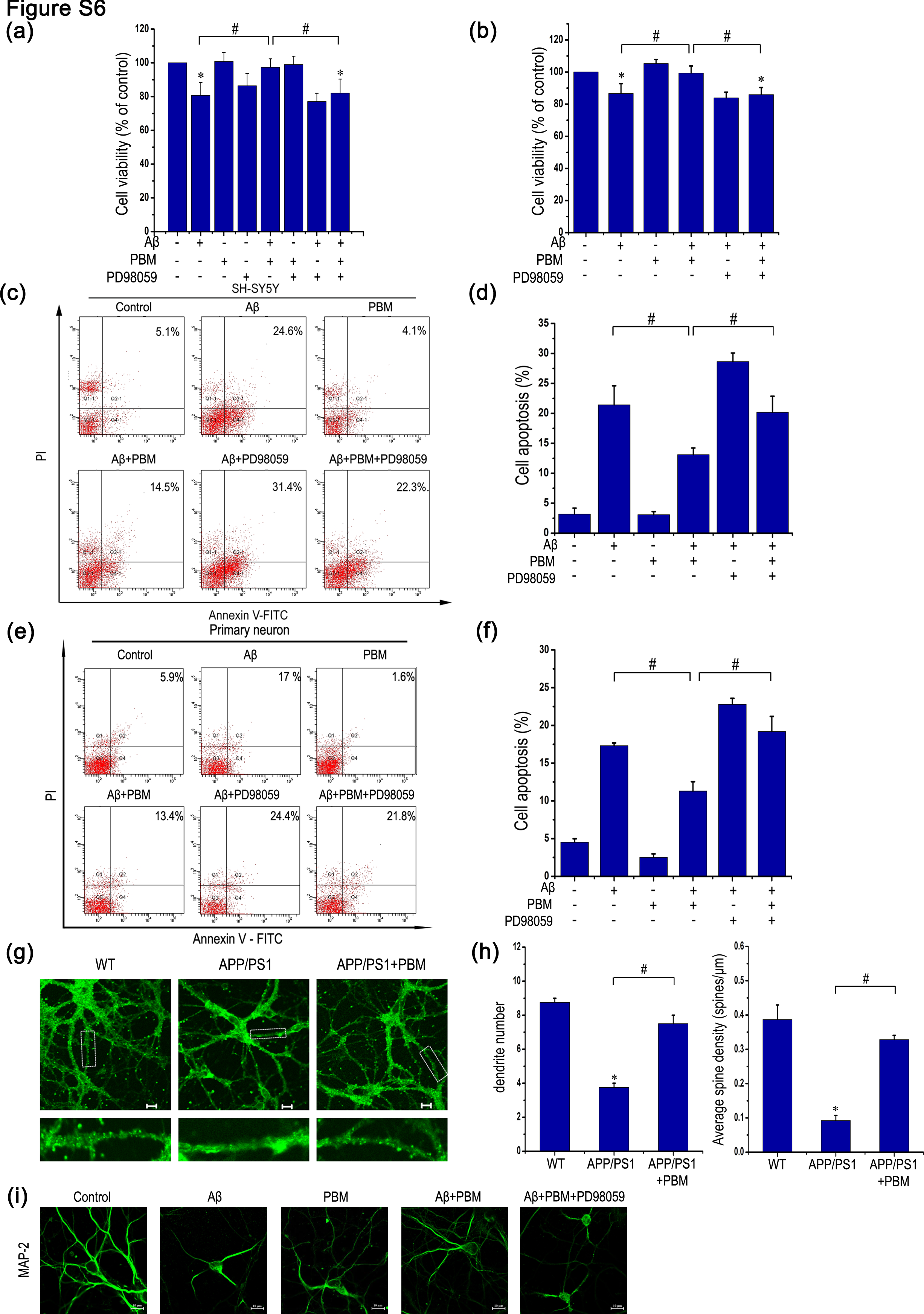


**Figure S6.** PBM treatment against Aβ-induced neurotoxicity. (a-b) Cell viability was assessed by CCK-8 assay in SH-SY5Y cells and primary neurons treated with Aβ_1-42_ (1 μM) and/or PBM (2 J/cm^2^). All data are presented as means + SEM for 3-5 individual experiments; **p* < 0.05 vs. control group, #*p* < 0.05 vs. indicated group by One-way ANOVA. (c-d) SH-SY5Y cells treated with Aβ_1-42_ and/or PBM were double stained with apoptosis markers Annexin V/PI using flow cytometric analysis (at least three individual experiments, mean + SEM, One-way ANOVA, #*p <* 0.05 vs. indicated group). (e-f) Primary neurons treated with Aβ_1-42_ and/or PBM were double-stained with apoptosis markers AnnexinV/PI using flow cytometric analysis (3-4 individual experiments were performed, mean + SEM, One-way ANOVA, #*p* < 0.05 vs. indicated group). (g) Representative photomicrographs of FITC-phalloidin labeling in primary neurons derived from APP/PS1 mice on 14 DIV under the treatment with or without PBM. Scale bar, 10 μm. (h) Quantification of dendrite number and spine density under indicated treatments. For each group, > 15 neurons were measured. All data are presented as means + SEM for at least three individual experiments. **p <* 0.05 vs. control group; #*p <* 0.05 vs. indicated group by Two-way ANOVA. (i) Representative photomicrographs of MAP-2 labeling in primary neurons under treatment with Aβ_1-42_ and/or PBM in the presence of PD98059. Scale bar: 10 μm.


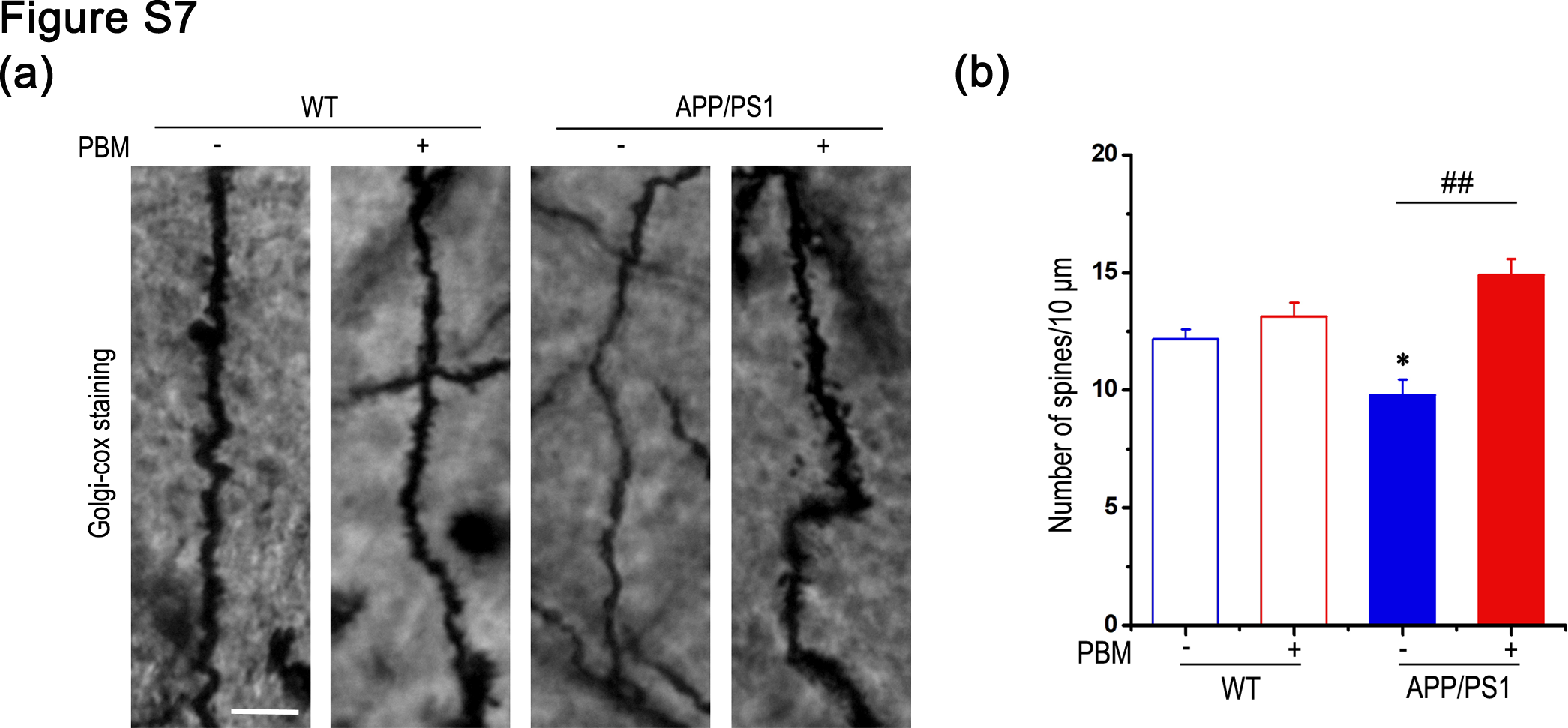


**Figure S7.** The effect of PBM on dendritic spine density of APP/PS1 mice by Golgi-Cox staining. (a) Representative images of Golgi-Cox staining taken from cortex region of each group (scale bar: 10 μm). (b) Quantification of spines per 10 μm in WT and APP/PS1 mice with or without PBM treatment (*n* = 5 mice for each group, mean + SEM, Two-way ANOVA, **p* < 0.05 vs. WT group; ##*p <* 0.01 vs. indicated group).


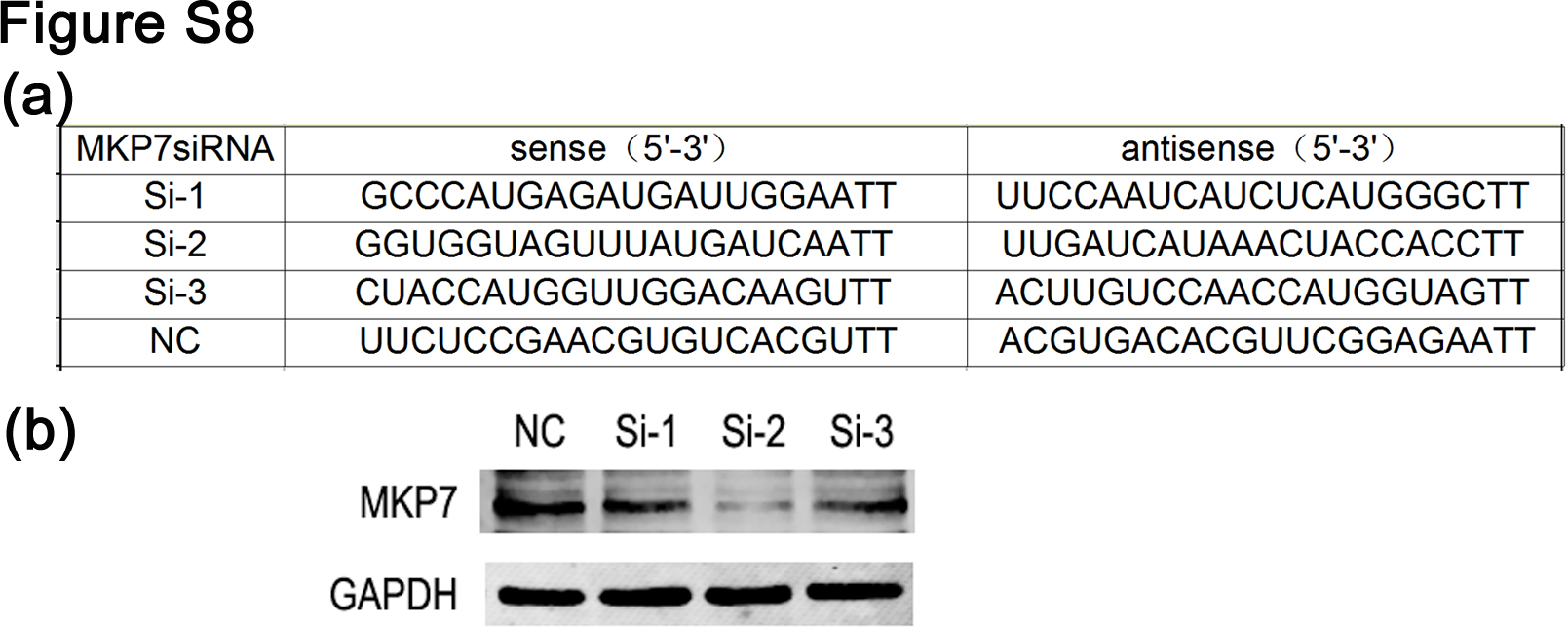


**Figure S8.** The siRNA interference experiments of MKP7. (a) siRNAs for MKP7 used in this study. (b) The interference efficiency was detected by western blot. The results were obtained from 3-4 independent experiments.


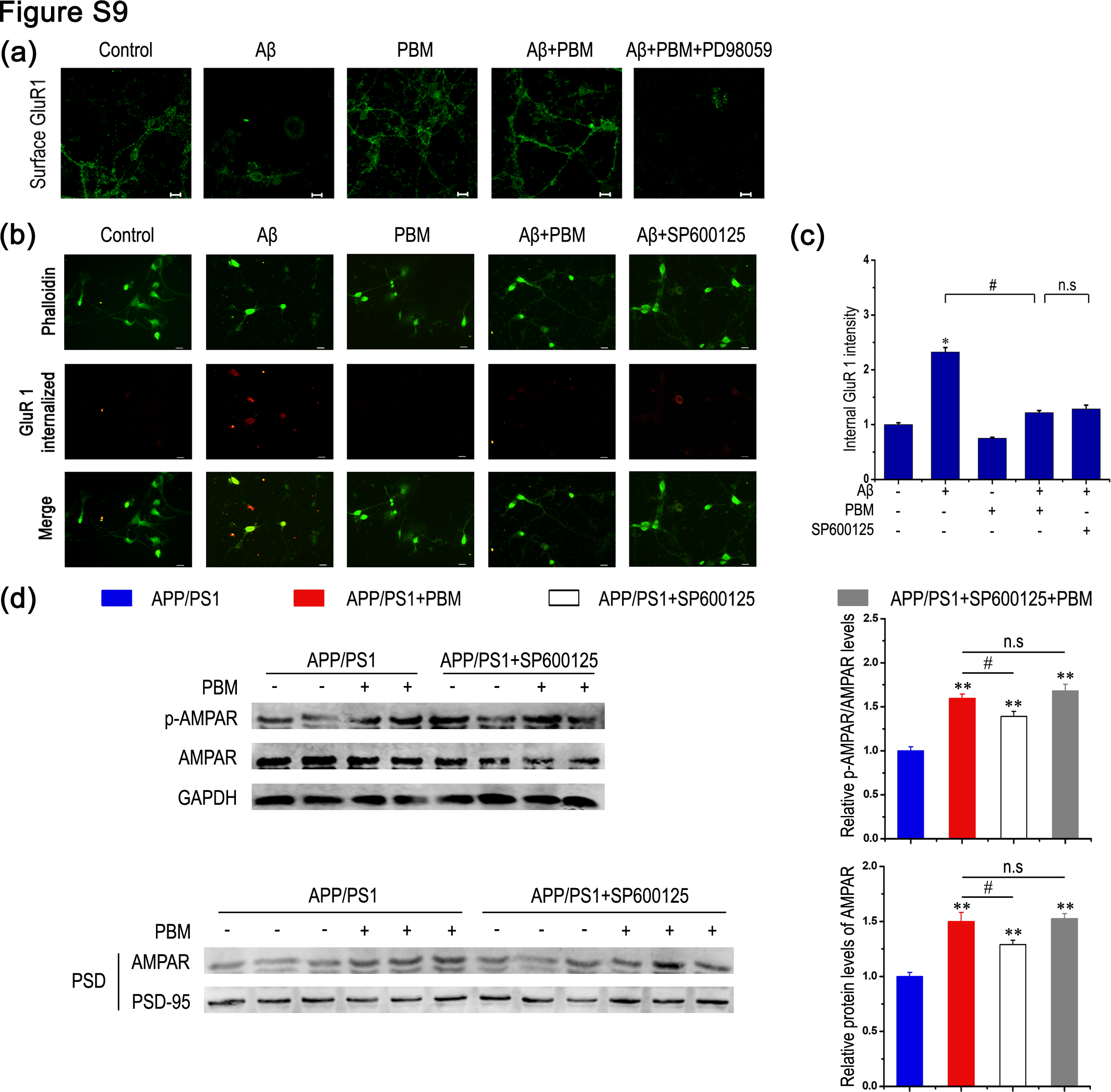


**Figure S9.** PBM attenuates endocytosis of synaptic AMPA receptors. (a) Representative confocal microscopy of surface GluR1 staining in response to different treatments. Scale bar, 10 μm. (b-c) To assay total AMPAR endocytosis, surface AMPARs were labeled in live neurons by 15 min incubation at 37^o^C with an antibody directed against the N-terminus of the GluR 1 subunit. After washout of the antibody, cells were treated with Aβ_1-42_ and/or PBM, antibodies remaining on surface receptors were stripped with an acidic solution. These experiments show that antibodies used for labeling internalized AMPARs does not label surface AMPARs. Representative images (b) and Quantitation (c) of total endocytosis of AMPARs (GluR1 internalized) in primary neurons stimulated with various treatments. Scale bar, 40 μm. (d) Representative western blot assay of p-AMPAR, and AMPAR, and immunoblots of PSD proteins from APP/PS1 mice treated with PBM and/or SP600125 (*n* = 5 for each group). All data are presented as means + SEM for at least three individual experiments, One-way/Two-way ANOVA, **p <* 0.05 vs. control group; ***p <* 0.01 vs. control group; #*p <* 0.05 vs. indicated group; n.s indicates *p* > 0.05.


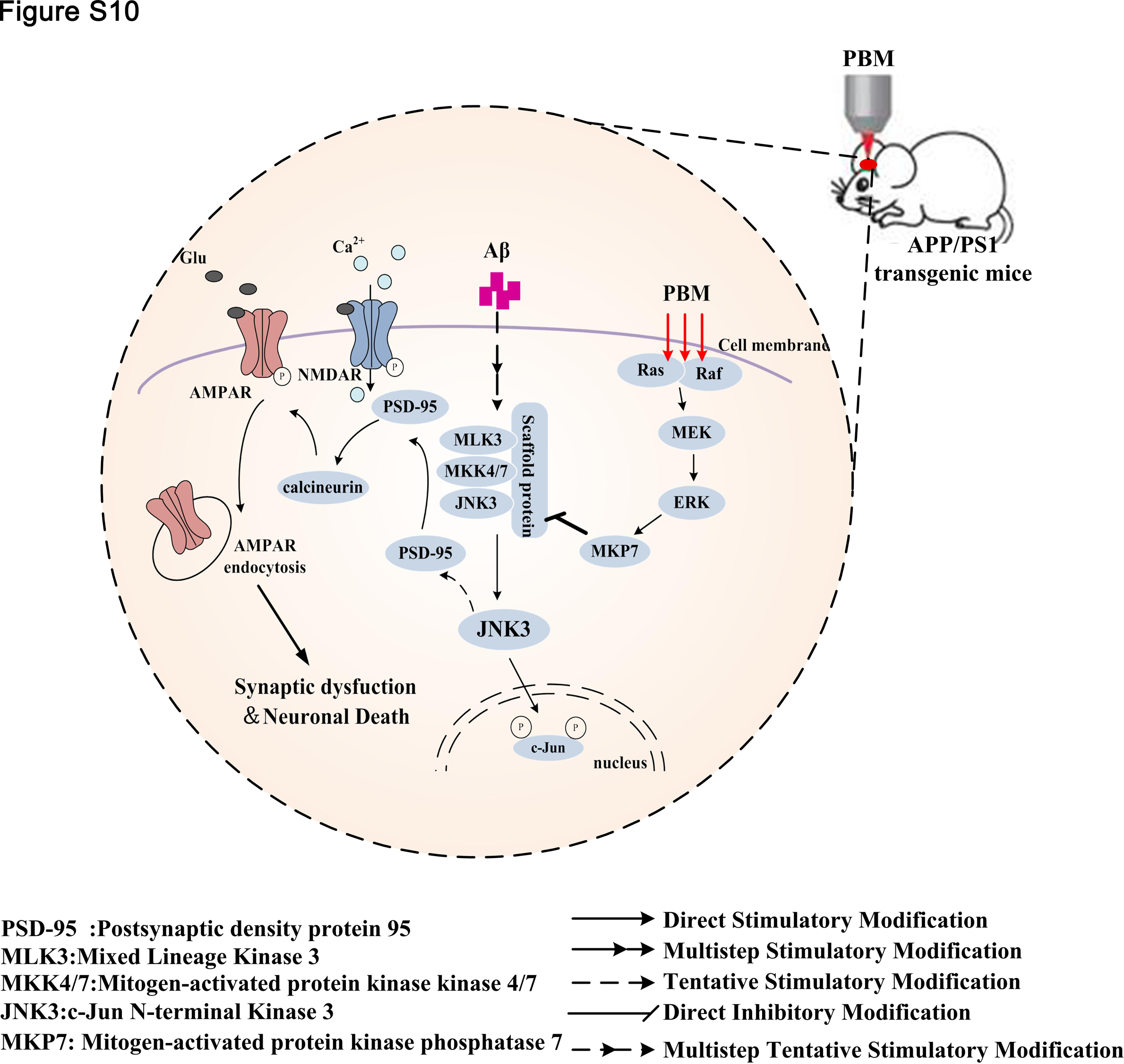


**Figure S10.** Schematic representation of the signaling pathway by which PBM attenuates synaptic dysfunction and neuronal death in APP/PS1 transgenic mice.
